# Sensorimotor integration, nutrition and gut microbiota in Ecuadorian autistic children – “Proyecto Wiñay”: a research protocol for a comparative cross-sectional study

**DOI:** 10.3389/fpsyt.2026.1721567

**Published:** 2026-04-30

**Authors:** Xiana Yago, Igor Eduardo Astudillo-Skliarova, Andrés Fernando Vinueza-Veloz, Dario Javier Guerrero-Vaca, Andrés Carrazco-Montalvo, Jefferson Santiago Piedra-Andrade, Jiří Svozilík, Sarita Lucila Betancourt-Ortiz, Sandra Victoria Abril-Ulloa, Cornelis P. Boele, Seth Sherry, Kayleigh Danielle Gultig, Henk-Jan Boele, Tannia Valeria Carpio-Arias, Maria Fernanda Vinueza-Veloz

**Affiliations:** 1 Independent researcher, Valencia, Spain; 2 Carrera de Nutrición y Dietética, Facultad de Salud Pública, Escuela Superior Politécnica de Chimborazo, Riobamba, Ecuador; 3 Carrera de Medicina, Facultad de Salud Pública, Escuela Superior Politécnica de Chimborazo, Riobamba, Ecuador; 4 Grupo de Investigación en Alimentación y Nutrición Humana (GIANH), Facultad de Salud Pública, Escuela Superior Politécnica de Chimborazo, Riobamba, Ecuador; 5 Centro de Referencia Nacional de Genómica, Secuenciación y Bioinformática, Instituto Nacional de Investigación en Salud Pública “Leopoldo Izquieta Pérez”, Quito, Ecuador; 6 Departamento de Biología Molecular, Centro de Biología Molecular Severo Ochoa (CSIC-UAM), Universidad Autónoma de Madrid, Madrid, Spain; 7 Escuela de Posgrado, Facultad de Ciencias de la Salud, Universidad de las Américas, Quito, Ecuador; 8 Carrera de Física, Maestría en Física, Facultad de Ciencias, Escuela Superior Politécnica de Chimborazo, Riobamba, Ecuador; 9 Grupo de investigación “Centro Politécnico de Investigación de Alimentos para el Desarrollo (CEPIAD)”, Facultad de Salud Pública, Escuela Superior Politécnica de Chimborazo, Riobamba, Ecuador; 10 Grupo de Investigación “Salud Pública, Alimentación y Actividad Física en el ciclo de la vida”, Carrera de Nutrición y Dietética, Facultad de Ciencias Médicas, Universidad de Cuenca, Cuenca, Ecuador; 11 BlinkLab Ltd., Perth, WA, Australia; 12 Neuroscience Institute, Princeton University, Princeton, NJ, United States; 13 Department of Community Medicine and Global Health, Institute of Health and Society, University of Oslo, Oslo, Norway; 14 Department of Child Health and Development, Norwegian Institute of Public Health, Oslo, Norway

**Keywords:** autism, children, Ecuador, gut microbiota, malnutrition, neurobehavior, sensorimotor

## Abstract

**Background:**

Autistic children experience disproportionately high rates of malnutrition compared to non-autistic (NA) peers. Sensorimotor integration differences (SMD), prevalent in 69–95% of autistic individuals, are hypothesized to drive this disparity. SMD contributes to core autistic traits that exacerbate feeding difficulties (e.g., food selectivity), potentially leading to malnutrition and gut dysbiosis. Current caregiver-reported SMD assessments lack validity in autistic populations and are prone to cultural and socioeconomic biases, limiting insight into nutritional outcomes.

**Objective:**

To investigate relationships between objectively measured SMD, dietary patterns, nutritional status, and gut microbiota composition in autistic children versus NA peers.

**Methods:**

This comparative cross-sectional study will recruit 100 autistic children and adolescents (aged 3–17 years) and 200 age-/sex-matched NA peers (including 100 with developmental disorders other than autism). Autism severity will be assessed using the Childhood Autism Rating Scale; NA peers will be screened with the Autism Spectrum Quotient-10. Dietary intake will be evaluated via three 24-hour dietary recalls, supplemented by questionnaires on socioeconomic status, health, and medications. Nutritional status (anthropometry, bioimpedance) and gut microbiota composition and diversity will be assessed. SMD proxies (sensory gating, sensory adaptation) will be measured using a smartphone application.

**Analysis:**

Group comparisons will employ multivariate regression models, adjusting for age, sex, socioeconomic status and medication use. We hypothesize significant between-group differences in dietary patterns, nutritional status, microbiota profiles, and SMD measures. Objectively measured SMD in autistic children and adolescents is expected to correlate with symptom severity, dietary patterns, and differences in microbiota composition and diversity.

**Ethics and dissemination:**

The study has been approved by the Escuela Superior Politécnica de Chimborazo Ethics Committee (Ref: IO-01-CEISH-ESPOCH-2025). All participants will receive personalized nutritional counseling. Findings will be disseminated through peer-reviewed open-access publications, scientific conferences, and community workshops.

## 1 Introduction

Autism is a neurodevelopmental condition characterized by two core diagnostic features, as defined by the Diagnostic and Statistical Manual of Mental Disorders (DSM-5): i) persistent deficits in social communication and interaction, manifesting as impairments in emotional reciprocity, verbal and nonverbal communication, and developing and maintaining relationships; and ii) restricted, repetitive patterns of behavior, interests, or activities, alongside cognitive inflexibility (1). These symptoms typically emerge in early childhood and cause significant functional impairment across social, occupational, and educational domains (2). In Ecuador, the estimated prevalence of autism is 682.70 cases per 100,000 individuals (3).

Although behavioral and cognitive impairments dominate autistic symptomatology, concomitant conditions such as malnutrition are prevalent. For example, studies indicate that overweight affects up to 35% and undernutrition up 20% of autistic children (3). Furthermore, autistic individuals are at higher risk of malnutrition, whether underweight, overweight, or obese, compared to non-autistic (NA) peers (4, 5). A critical driver of these disparities is sensorimotor integration differences (SMD), which alters responses to sensory stimuli (e.g., tactile, auditory, gustatory) in 69–95% of autistic individuals (6, 7).

Beyond food-related sensitivities (e.g., aversion to specific smells, tastes, textures), SMD contributes to malnutrition by underpinning core autistic traits including stereotypical behaviors, reduced behavioral flexibility, hyper- or hypo-reactivity, and anxiety. These traits lead to feeding problems such as food neophobia, heightened food selectivity, pica, and rumination (8). Consequently, individuals may suffer from deficiencies in essential nutrients like protein, iron, and vitamins, while consuming excessive amounts of polyunsaturated fats compared to their NA peers (8, 9). Such nutritional imbalances can further worsen core autistic symptoms and increase the risk of chronic conditions later in life (3, 10).

Restricted dietary patterns associated with SMD also impact the diversity, composition, and functionality of the gut microbiota (GM). Autistic children often show increased levels of taxa such as *Bacteroides*, *Parabacteroides*, *Clostridium*, *Faecalibacterium*, and *Phascolarctobacterium* and reduced levels of *Coprococcus* and *Bifidobacterium* (11, 12). Recent evidence further shows that microbial profiles vary with nutritional status (12). Among children with excess weight, those with autism have lower levels of *Roseburia* and *Faecalibacterium* and higher levels of *Eubacterium* and *Flavonifractor* than their NA peers (12). These dysbiotic shifts disrupt microbial balance, affecting the production of key microbial metabolites, including short-chain fatty acids (e.g., butyrate, acetate, propionate), neurotransmitters (e.g., gamma-aminobutyric acid), and vitamins.

Altered availability of these compounds may worsen autistic-related cognitive and behavioral symptoms by impairing the function of the gut-brain axis (13). For example, decreased levels of short-chain fatty acids have been linked to dysregulated serotonin signaling, which is often correlated with symptom severity (14). Furthermore, the increased prevalence of pathogenic bacteria associated with dysbiosis has been implicated in intestinal inflammation and hyperpermeability, which is referred to as “leaky gut” (15). These conditions can facilitate the translocation of bacterial and fungal harmful metabolites into the bloodstream and, later, into the central nervous system, thus further contributing to autistic symptomatology (16).

A major limitation in understanding the SMD-nutrition-microbiota relationship has been the reliance on caregiver-reported SMD assessments, such as the Short Sensory Profile (SSP). While instruments like the SSP have been extensively used in autism research, they remain subjective measures that may be influenced by caregiver perception, cultural context, and socioeconomic factors (17, 18). Moreover, their performance in non-Western populations has not been thoroughly evaluated. Neurometric tools such as pre-pulse inhibition of the startle response (PPI), offer an objective alternative for measuring sensorimotor assessment in autistic cohorts (19, 20). Innovations, such as a smartphone-based tool that enables accessible, non-invasive at-home neurobehavioral assessments, can provide standardized tests for measuring sensory gating and adaptation through the assessment of PPI and startle habituation (SH), as well as general sensorimotor behaviors (21, 22).

Therefore, we hypothesize that SMD in autistic children is associated with imbalanced dietary patterns, leading to malnutrition and changes in GM diversity and composition. These physiological alterations may collectively exacerbate autistic symptom severity, which in turn could further intensify SMD and feeding difficulties, establishing a self-perpetuating cycle of worsening symptoms and malnutrition. Our main objective is to investigate the relationships between SMD, dietary patterns, nutritional status and GM characteristics in autistic children compared to NA peers. Specifically, the study will (1): Compare dietary patterns and nutritional status between autistic children and NA peers (2). Assess differences in the GM (diversity, composition) between autistic children and NA peers (3). Quantify SMD by assessing PPI and SH and compare profiles across autistic children and NA peers (4). Examine associations between SMD, dietary patterns, nutritional status, GM profiles, and autistic symptom severity (5). Explore whether SMD mediates the relationship between autism and adverse nutritional/GM outcomes.

## 2 Methods and analysis

### 2.1 Study design and context

The present document outlines the research protocol for an analytical observational, comparative cross-sectional study. The study will be conducted in the cities of Quito and Riobamba, which are located in the Highlands of Ecuador. Data collection is planned to start in the fourth trimester of 2025 and end the fourth trimester of 2026. Data analysis is planned to start in the first trimester of 2026 and end the fourth trimester of 2028. A pilot study involving 10 autistic and NA children will be conducted during the third trimester of 2025 to refine procedures and instruments. The overall study design and procedures are summarized in **Supplementary Figure 1**.

### 2.2 Study population, selection criteria and sample size

#### 2.2.1 Group A - autistic children

This group includes children and adolescents aged 3 to 17 years (n = 100), from diverse socioeconomic backgrounds, who have been previously diagnosed with autism by certified physicians following the Ecuadorian Clinical Practice Guide and was based on the DSM-5 criteria (23). At least 100 participants are required, but we will accept up to 150 children.

*Inclusion criteria*

- Age between 3 and 17 years.
- Previous autism diagnosis by a neurologist or psychiatrist after meeting the DSM-5 diagnostic criteria for autism. Such diagnosis will include the following International Classification of Diseases, 10th Edition (ICD-10) categories: F84.0 (Childhood autism), F84.5 (Asperger’s syndrome), F84.8 (Other pervasive developmental disorders), and F84.9 (Pervasive developmental disorder, unspecified).

*Exclusion criteria*

- Inability to sit and/or stand independently.

#### 2.2.2 Group B - non-autistic children without neurodevelopmental disorders

This group will include children and adolescents aged 3 to 17 years (n = 100), from the general population with age and gender distribution similar to the autistic group.

*Inclusion criteria*

- Age between 3 and 17 years.

*Exclusion criteria*

- A 10-item Autism Spectrum Quotient (AQ-10) score of 6 points or higher.
- A Parent’s Observations of Social Interactions (POSI) score of 3 points or higher.
- History of diagnosis, therapy, or treatment for any neurodevelopmental disorder coded under ICD-10 F84.
- Inability to sit and/or stand independently.

#### 2.2.3 Group C - non-autistic children with other neurodevelopmental disorders

This group will include children and adolescents aged 3 to 17 years (n = 100) diagnosed with intellectual, psychological, emotional, or behavioral developmental disorders not including autism.

*Inclusion criteria*

- Age between 3 and 17 years.
- Diagnosis with psychological developmental disorders (ICD-10 F80-F89), which includes: F80 (specific developmental disorders of speech and language), F81 (specific developmental disorders of school skills), F82 (specific developmental disorder of motor function), F83 (mixed specific developmental disorders), F88 (other disorders of psychological development) and F89 (unspecified disorder of psychological development). Diagnosis with intellectual disabilities (ICD-10 F70-F79), including F70 (mild mental retardation), F71 (moderate mental retardation), F78 (other mental retardation), F79 (unspecified mental retardation). Diagnosis with emotional and behavioral disorders usually occurring in childhood and adolescence (ICD-10 F90-F98), which includes F90 (hyperkinetic disorders), F91 (conduct disorders), F92 (mixed disorders of conduct and emotions), F93 (emotional disorders with onset specific to childhood), F94 (disorders of social functioning with onset specific to childhood), F95 (tic disorders), and F98 (other emotional and behavioral disorders that commonly occur in childhood and adolescence).

*Exclusion criteria*

- AQ-10 score of 6 points or higher.
- POSI score of 3 points or higher.
- Diagnosis with or report of having received therapy or treatment for a neurodevelopmental disorder coded as ICD-10 F84.
- Being unable to sit and/or stand autonomously.

#### 2.2.4 Sample size

The sample size was determined based on the expected difference in malnutrition prevalence between autistic children and their NA peers in a cross-sectional comparison. According to the *Encuesta Nacional de Salud y Nutrición* (24), 35% of the Ecuadorian children younger than 11 years of age are overweight or obese, while among adolescents the prevalence of overweight or obesity is 30%. The prevalence of extreme thinness is 2% among Ecuadorian children and adolescents. Based on these figures, we assumed an approximate malnutrition prevalence of 37% in the NA population.

There is no published data on prevalence of malnutrition in Ecuadorian autistic children up to date. To estimate this value, we referred to data from a recent study in another Latin American country (Mexico), which reported a malnutrition prevalence of 57.50% among autistic children (45% obese/overweight, and 12.50% underweight) (25). To estimate the sample size required to detect this difference (~20.50%) between the groups, we used the formula for comparing two independent proportions in a two-sided hypothesis test (26):

$n=\frac{{\left(Z_{\alpha /2}+Z_{\beta }\right)}^{2}\times [p_{1}(1-p_{1})+p_{2}(1-p_{2})]}{{\left(p_{1}-p_{2}\right)}^{2}}$, where:

$Z_{\alpha /2}$ = 1.96 (corresponding to a 95% confidence level),

$Z_{\beta }$ = 0.84 (corresponding to 80% power),

$p_{1}$ = 0.57 (estimated malnutrition prevalence in autistic children - Group A),

$p_{2}$ = 0.37 (estimated malnutrition prevalence in NA children - Group B).

The formula yields an approximate sample size of 94 participants per group. To account for a projected non-response rate of 5%, the sample size increases to 100 participants per group. This sample size is also adequate for detecting large effect sizes in secondary comparisons, such as the prevalence of SMD, which has been reported to be between 81.50% and 95.00% among autistic children and may reach 16.50% in children of the population (7, 18, 27). Thus, our sample will be sufficient for the validation of the neurometric test, achieving a statistical power close to 1.

### 2.3 Recruitment and patient involvement

This study protocol was designed in collaboration with a special education school in Quito and a child neurodevelopment center in Riobamba. Both are private centers where autistic children and adolescents receive rehabilitation services. Additional participants will be recruited through posters and flyers distributed on social networks, at educational institutions, and through associations of autistic children’s parents. Children in Group B will be recruited from the family networks of Group A participants and matched for sex and age with their autistic peers. The target ratio between Groups A and B is approximately 1:2. During the development of the protocol, parents and professionals from recruitment sites provided valuable recommendations about the recruitment process, procedures and study goal.

### 2.4 Procedures

Data will be collected through three ~60-minute visits conducted at participants’ homes or preferred locations by trained researchers (including medical professionals). All personnel will receive standardized training in instrument administration, pediatric assessment protocols, and transcription procedures. A summary of visits is depicted in **Table 1**.

**Table 1:** Summary of study visits and procedures.

Visit	Procedures conducted	Measurements/tools used
Visit 1	- Informed consent - Sociodemographic/clinical data - First dietary assessment - Autism screening - Handing out stool sample collection kits	- Questionnaires - CARS (group A) - POSI/AQ-10 (all groups) - 24-HDR
Visit 2	- First neurobehavioral assessment - Anthropometry/bioimpedance - First dietary assessment	- Smartphone application - Stadiometer, InBody120 - 24-HDR
Visit 3	- Second neurobehavioral assessment - First dietary assessment - Stool sample collection - Completion of pending data	- Smartphone application - 24-HDR

24HDR, 24-hour dietary recall; AQ-10, 10-item Autism Spectrum Quotient; CARS, Childhood Autism Rating Scale; POSI, Parent’s Observations of Social Interactions.

The following data will be collected:

- Sociodemographic and medical information: Personal data will be collected using a customized questionnaire (Annex1). Socioeconomic status information will be obtained using the *Encuesta de Estratificación del Nivel Socioeconómico*, elaborated by the *Instituto Ecuatoriano de Estadísticas y Censos* (Annex 2). General health status, treatments, and therapies will be documented using caregiver-directed questionnaires (Annex 3).
- Nutritional assessment: Dietary patterns will be assessed through three 24-hour dietary recall (24-HDR) assessments on three non-consecutive days (Annex 4 of the **Supplementary Material**). Nutritional status will be evaluated via anthropometric measurements and body composition analysis using bioelectrical impedance, documented in a technical sheet (Annex 5 of the **Supplementary Material**). Caregiver and healthcare professional perceptions regarding nutrition will be captured through semi-structured interviews (Annex 6 of the **Supplementary Material**).
- Neuropsychological assessment: Autism symptom screening will be conducted using screening tools for different ages: children <4 years will be assessed using the POSI (Annex 7 of the **Supplementary Material**), those ages 4–11 years with the AQ-10 child version (Annex 8 of the **Supplementary Material**), and those ages >11 years with the AQ-10 adolescent version (Annex 9 of the **Supplementary Material**). Symptom severity will be evaluated exclusively in Group A using the Childhood Autism Rating Scale (CARS) (Annex 10 of the **Supplementary Material**).
- Neurophysiological Assessment: Sensorimotor integration differences will be assessed using a smartphone application.
- GM analysis: The composition and diversity of the GM will be analyzed through stool sample collection and bacterial DNA sequencing.

The initial session will involve obtaining informed consent, collecting baseline sociodemographic information, health status, treatments/therapies, and conducting the first 24-HDR. CARS and autism screening questionnaires (POSI, AQ-10) will be administered concurrently. Caregivers will receive stool collection kits with instructions; child presence is optional. During the second session, children will undergo the first neurobehavioral assessment (15–20 minutes), comprehensive anthropometric measurements, body composition evaluation, and the second 24-HDR. The third session will include another neurobehavioral assessment, the final 24-HDR, and stool sample collection. Any outstanding data will be collected in the last session.

Throughout the study, caregivers may contact researchers for clarification and will have the option to complete questionnaires remotely. All participants retain the right to withdraw at any time without penalty. Following the study’s completion, caregivers will receive individualized reports on their child’s nutritional status, along with a summary of the aggregate study results. All materials will be presented in an accessible format and in Spanish.

### 3.5 Assessments

#### 3.5.1 Sociodemographic and medical information

Sociodemographic and clinical data will be collected through a customized survey directed to the caregivers. The variables include age, sex assigned at birth, socioeconomic position, medication use, and general health status. Age will be recorded in years and months, calculated as the time elapsed from birth to the date of the first interview with the field researchers.

The socioeconomic position of children will be evaluated using a previously described instrument (28). Briefly, the instrument evaluates six dimensions: (i) Housing characteristics; (ii) access to technology; (iii) possession of material goods; (iv) consumption habits; (v) level of education of the head of the household; and (vi) household income. The sum of the scores for all questions ranges from 0 to 1000. According to this score, households are classified into five socioeconomic groups: A (>845), high; B (>696 and ≤ 845), upper-middle; C+ (>535 and ≤ 696), middle; C− (>316 and ≤535), lower-middle; and D (≤316), low.

#### 3.5.2 Neuropsychological assessment

All participants will be screened with age-appropriate instruments. Children under 4 years will be assessed using the POSI (29), those aged 4–11 years with the AQ-10 child version, and those 12 years and older with the AQ-10 adolescent version (30). Additionally, children in Group A will undergo a formal evaluation of autism symptom severity using the Spanish version of the Childhood Autism Rating Scale (CARS) (31, 32).

##### 3.5.2.1 Parent’s observations of social interactions

POSI is a screening tool for neurotypical children aged 16 to 48 months, administered to parents or immediate caregivers. The reported internal consistency is high (α = 0.83), with a sensitivity of 89% and a specificity of 54% (29). This instrument is a brief (7-item) questionnaire based on parental observations of social interactions, the purpose of which is to identify possible signs of autism. Each item is scored as a 0 or 1; a total score of 3 or higher indicates autistic traits.

##### 3.5.2.2 10-item autism spectrum quotient

The AQ-10 is a screening tool designed to measure autistic associated traits. There are adapted versions for different age groups; for this research, the child version, for ages 4 to 11, and the adolescent version, for ages 12 and up, will be used (31). The original AQ consists of 50 questions, but we will use an abbreviated version consisting of 10 items. AQ-10 evaluates various areas, including interpersonal relationships, social skills, and communication. AQ-10 scores range from 0 to 10 points, with scores of 6 and above indicating autistic traits. In the validation study by Allison et al. (2012), the AQ-10 child version showed a sensitivity of 95% and a specificity of 97%, while the AQ-10 adolescent version showed a sensitivity of 93% and a specificity of 95% for the ≥ 6 cutoff.

##### 3.5.2.3 Childhood autism rating scale

CARS will be administered only to autistic children (Group B) to evaluate the severity of autistic symptoms. This scale is a diagnostic observational assessment that has consistently demonstrated strong psychometric properties. In the original validity study, the internal consistency was exceedingly high (Cronbach’s alpha α = .94) (33). In the Spanish version, the evaluator assigns a score from 1 to 4 in each of the 15 areas included (31, 32). A score of 1 is given when age-appropriate behavior is observed, while a score of 4 is given when the observed behavior is at the farthest end of what is expected for the child’s age. Scores equal to or greater than 30 are indicative of autism. Specifically, scores between 30 and 36.5 are classified as mild to moderate autism, while scores equal to or greater than 37 indicate severe autism.

##### 3.5.2.4 Note on screening results

If a score meets or exceeds the cutoff on any screening tool (POSI or AQ-10), parents or caregivers will be informed that the result suggests a suspicion of autism. They will be advised that these are screening instruments with the potential for false positives and negatives, and that the result does not constitute a definitive diagnosis. A confirmation by a qualified professional will be recommended.

#### 3.5.3 Nutritional assessment

##### 3.5.3.1 Anthropometry

Height and weight will be measured in duplicate by trained personnel applying standardized protocols. During the assessment children are barefoot and wear light clothing. Body weight will be measured using a digital scale (Seca) calibrated to the nearest 0.1 kilograms (kg). Height measured in centimeters (cm)) will be assessed using a portable stadiometer (Seca) with a precision of 1millimetre (mm). Waist circumference will be measured midway between the lowest rib margin and the iliac crest, with the children in a standing position.

A non-elastic flexible SECA measuring tape will be used for measuring circumferences. Neck circumference (measured in cm) will be measured using a non-stretchable measuring tape to the nearest 0.10 cm. The tape will be placed on the midline of the neck between the cervical backbone and the anterior neck while the children stand upright, face forward, and shoulders relaxed. Waist circumference (measured in cm) will be measured at the midpoint between the lowest border of the rib cage and the upper iliac crest to the nearest 0.1 cm. Arm circumference will be measured at a mark point in the middle of the posterior surface of the left humerus between the acromion and the olecranon process.

Abdominal obesity will be defined as having a waist circumference at or above the 90th percentile in comparison to children of similar age and sex. Percentile cut-off points for abdominal circumference will be based on the reference data from African-American, European-American, and Mexican-American children (34). Cut-off points for arm circumference will be ≥ 13.6 cm (normal), ≤ 13.5 to ≥ 11.6 cm (undernutrition), and ≤ 11.5 cm (severe undernutrition) (35). Filgueiras parameters will be used for determining malnutrition in children using neck circumference (36).

Using the World Health Organization (WHO) software Anthro and AntroPlus, we will calculate z-scores for BMI-for-age, and height-for-age. Anthro uses 2006 WHO growth reference curves (37). AntroPlus uses the 2007 WHO growth reference curves (38). The standard deviation (SD) cut-off points we will use are the following: BMI-for-age (< -2 SD, thinness; < -3 SD, severe thinness; > +1 SD, overweight; > +2 SD, obesity); height-for-age (> +2 SD, tall; +2 SD to -2 SD, normal; < -2 SD, stunting).

##### 3.5.3.2 Bio-impedance

Body composition will be measured using bioelectrical impedance analysis (InBody). Children will stand on the InBody scale with their bare hands and feet making contact with the eight electrodes (2 palm, 2 thumb, 2 toe, and 2 heel); the measurement will take approximately 30 seconds. Body composition measurements will include: Total body fat percentage, visceral fat percentage, body water measured in liters, and muscle mass percentage. Measures will be automatically calculated by InBody (39, 40).

##### 3.5.3.3 24-hour dietary recall

Data on recent food intake will be collected using 24-HDR, a previously validated instrument on three different days (two weekdays and one weekend day) (41). Data collection will include information on the time of food consumption, type, quality and quantity of food (using home measurements), and cooking method. The foods consumed will be classified into gluten sources, casein sources, and ultra-processed foods. A single food item may be assigned to more than one category (42). Visual food models and photographs using local food albums will be used to accurately estimate the quantities of food consumed (43). Total calories, grams of protein, fat, and carbohydrate, intakes of iron, calcium, folate, vitamin C, and vitamin D will be calculated using food composition reference tables (44). The detailed food-level data obtained from the 24-hour dietary recalls will also allow us to examine dietary patterns by food groups (e.g., dairy, fruits, cereals, fish), following recent recommendations in the literature (45).

#### 3.5.4 Neurophysiological assessment

Neurobehavioral assessments will be performed using a smartphone-based platform (21). The assessment will include a general measurement of spontaneous and stimulus-evoked postural, head, facial, and vocal responses, along with specific neurometric tests, including PPI and SH. During the experiment, children will use wired headphones to watch an audio-normalized video on a smartphone. The application delivers auditory stimuli, and the participant’s responses are captured using the smartphone’s front-facing camera at 60 frames per second.

Two audio-normalized videos will be used: one for younger children (aged 3–5 years), featuring a compilation of moving shapes and animals, and one for older children (aged 6 years and older), which depicts a basic storyline of a boy and a girl playing a video game. Both videos are devoid of any language content. The videos are audio-normalized by adjusting the audio track to ensure its peak level does not exceed approximately 60–70 decibels (dB).

While the participant watches the video, computer vision algorithms are used to track and record the position of the participant’s facial landmarks over time. The individual eyeblink traces will be analyzed with custom software developed in R. Eyelid position is calculated based on the tracked facial landmarks. Eyeblink traces are filtered and normalized as previously described (22). From valid normalized trials, we will extract the following three outcome measures: (i) PPI, (ii) SH, and (iii) general motor behavior.

##### 3.5.4.1 Prepulse inhibition of acoustic startle response

PPI is the behavioral phenomenon whereby the magnitude of the startle response is inhibited when a short, loud startling sound (the pulse) is preceded by a weaker sound that does not itself elicit a startle reflex (the prepulse). The PPI training session will last approximately 12 minutes, contain 44 trials, and will be administered to all children. We will use a pulse consisting of a 105 dB, 50 ms white noise burst. The prepulse will consist of a 50 ms white noise burst of varying amplitude that is always softer than the pulse, delivered at three different intensities: 10% (soft), 25% (medium), and 40% (high) of the pulse amplitude. For the soft and high prepulse intensities, a fixed interstimulus interval (ISI) of 120 ms will be used between the prepulse and the pulse. For the medium prepulse intensity, ISIs of 60, 120, and 240 ms will be used. The session will begin with 2 practice trials containing white noise bursts at various soft intensities to allow the participant to relax and settle into the movie. Subsequently, 7 blocks of 6 trials will be presented. Each block will consist of one pulse-only trial and five prepulse-pulse trials. The order of trials will be randomized and will differ between blocks. The intertrial interval will be randomly set between 4 and 14 seconds.

*Dependent variables*

- Acoustic startle response (ASR) amplitude and variability: The normalized peak eyelid closure amplitude in response to the pulse-alone trials and its standard deviation.
- ASR latency to peak: The time from pulse onset to the peak of the eyeblink response.
- Prepulse-elicited response amplitude: The normalized peak eyelid closure amplitude in response to the prepulse-alone.
- Prepulse-elicited response latency: The time from prepulse onset to the peak of the eyeblink response it elicits.
- Prepulse-pulse response amplitude and variability: The normalized peak eyelid closure amplitude in response to the pulse when it is preceded by a prepulse, and its standard deviation.
- Percent PPI: The percentage inhibition of the ASR amplitude due to the prepulse, calculated as: % PPI = 100 - [(mean ASR amplitude on prepulse-pulse trials)/(mean ASR amplitude on pulse-alone trials) × 100].
- Prepulse excitation: A measure of hyper-responsiveness to the prepulse itself.
- Habituation: The change in normalized ASR amplitude to pulse-alone trials over the course of the entire session.

##### 3.5.4.2 Startle habituation

All children will complete a second neurobehavioral assessment session lasting approximately 12 minutes that contains 22 stimulus trials. The session will begin with two practice trials to allow participants to acclimate to the stimuli. The remaining 20 trials will consist of short-term habituation assessments. We will present six 50 ms, 105 dB white noise pulses, delivered in either a rhythmic or random interstimulus interval (ISI) pattern, as previously described (22).

*Dependent variables*

- Short-term habituation: The change in the normalized amplitude of reflexive eyeblinks between the first white noise pulse and the average of the subsequent five pulses, calculated for both random and rhythmic stimulus patterns.
- Cumulative response: The cumulative sum of the normalized eyeblink amplitude across all trials for both random and rhythmic stimulus patterns.
- Anticipatory blinks: The amplitude of eyeblinks occurring immediately (< 100 ms) prior to the onset of the pulse.

##### 3.5.4.3 General sensorimotor behavior

During the PPI and SH sessions, the smartphone’s front-facing camera and microphone passively capture a broad range of behavioral data, extending beyond the specific eyeblink responses required for the primary neurometric tests. Ten specific behaviors previously identified as differing between autistic and NA children will be analyzed in this study (22).

*Dependent variables:*

- Task engagement: The percentage of trials in which the participant is not detectable within the smartphone’s camera frame.
- Headphone interaction: The percentage of trials with observed touching or adjustment of the headphones.
- Non-syllabic vocalizations: The frequency (percentage of trials) and total duration of non-linguistic vocal sounds.
- Postural stability: The degree of anteroposterior postural sway.
- Head movement: The percentage of trials with observed head rotation.
- Orofacial movement: The frequency and duration of mouth openings.
- Oculomotor activity: The mean amplitude of horizontal pupil movement per session.
- Vestibulo-ocular reflex (VOR) function: The correlation between the direction of head turns and compensatory pupil movement.
- Initial baseline variability: The variation in baseline (pre-stimulus) eyelid position during the first five trials of a session.
- Baseline stability: The change in the variability of baseline eyelid position from the start to the end of the session.

#### 3.5.5 Microbiota assessment

For stool sample collection from children, parents will be provided with faecal collection containers and instructed to collect the sample immediately before delivery. Instructions for the parents will be provided in written format with animated graphics (Annex 11 of the **Supplementary Material**). Stool samples will be collected in an OMNIgene•GUT OM-200 (DNA Genotek Inc.; Ottawa, Canada) or a DANASTOOL (Danagen; Barcelona, Spain) tubes and transported to the laboratory facilities for their subsequent storage at -80 °C until further analysis.

The GM composition of all participants will be characterized through gene marker amplification and sequencing, which is a highly accurate and specific approach for profiling microbial communities. GM analysis will be performed as reported previously with a few modifications detailed in Annex 12 of the **Supplementary Material** (46). Briefly, this analysis will comprise four steps: DNA extraction, library preparation, sequencing and bioinformatics.

Genomic DNA (gDNA) will be extracted and purified from stool samples using a bead-beating-based approach. Library preparation will involve a PCR-amplification of the 16S rRNA V3 region, followed by the attachment of dual index barcode adapters. The indexed DNA will then be sequenced in paired-end reads of 300 bp using the Illumina MiSeq Platform. Finally, bioinformatics analysis will be performed using the DADA2 protocol (46, 47). Differences within (alpha-diversity) and between (beta-diversity) groups will be assessed using QIIME2 (48). Alpha-diversity will be quantified by Shannon diversity index, whereas beta-diversity will be evaluated using principal coordinate analysis based on Bray–Curtis dissimilarity matrices (49, 50).

#### 3.5.6 Interviews with caregivers and health professionals

The perceptions of caregivers and health personnel regarding the dietary patterns and health of children and adolescents with ASD will be investigated using a qualitative approach. We will extend invitations to all parents, primary caregivers, and health professionals in the specialized education centers participating in the “Proyecto Wiñay”. Face-to-face or videocall interviews will be scheduled with interested participants, depending on their availability. Approximately 20 in-depth interviews will be conducted with caregivers and health personnel or until information saturation (i.e., repetition, absence of novelty) is reached.

The interview is thematic and semi-structured, lasting between 30 minutes and one hour. It will be recorded to ensure a verbatim transcription and coding, which will anonymize participants. The analysis will focus on key categories: challenges and opportunities for healthy nutrition in children with autism, and challenges and opportunities in caring for children’s health. A thematic analysis of the collected information will be conducted using Atlas.ti software (v 24.1.1.30813) and an inductive approach (51). The analysis process consists of six steps: familiarization with the data, creation of initial codes, searching for themes, reviewing themes, defining and naming themes, and writing the report (52). The results of the thematic analysis will also be visualized as thematic networks (53).

### 3.6 Analysis plan

#### 3.6.1 Microbiota analysis

Homogeneity of multivariate dispersions will be tested using the *betadisper* function in the *vegan* R package (54). Differences in microbial community composition (beta-diversity) between children with ASD and their NA peers will be determined using permutational multivariate analysis of variance (PERMANOVA) through the ADONIS2 function, also in *vegan*. Pairwise comparisons will be performed using the Wilcoxon rank sum test with Benjamini-Hochberg p-value adjustment (55).

#### 3.6.2 Analysis of the relation between exposures and outcomes

In all analyses, the researchers will blind the identity of the comparison groups. Statistical analysis will be performed using multilevel mixed-effects linear and nonlinear models in R Studio and Python (56). These models offer several advantages over standard parametric and non-parametric tests: they are more robust to violations of normality, which is common in biological data, and they do not require homoscedasticity (57, 58). Additionally, they accommodate the nested structure of our data (trials nested within sessions, sessions nested within participants, participants nested within groups) and handle missing data more effectively than repeated-measures ANOVA. Normality of residuals will be assessed visually with quantile-quantile (Q-Q) plots and statistically using the Shapiro–Wilk test; homoscedasticity will be evaluated with the Breusch–Pagan test (59, 60). Data will be considered statistically significant at a p-value < 0.05. Given the large number of variables related to diet, nutritional status, ASD severity, and sensorimotor integration, exploratory analyses will include the Kruskal–Wallis test to compare groups on non-normally distributed variables (61). In addition, cluster analysis (k-means clustering and equivalent algorithms) will be used to identify profiles of gut microbiota or dietary patterns (62).

### 3.7 Ethics

This study protocol was approved by the Committee on Ethics in Human Research (CEISH) at the Escuela Superior Politécnica de Chimborazo (Ref No: IO-01-CEISH-ESPOCH-2025). The study will be conducted in accordance with the principles of the Declaration of Helsinki.

Prior to participation in “Proyecto Wiñay”, written informed consent will be obtained from the parents, guardians, or legal representatives of the children (see Annex 13 in the **Supplementary Material**). In cases where a non-emancipated minor lives under the care of grandparents, one grandparent serving as the legal guardian may provide consent. Adolescents aged 15 years or older will provide informed assent using a form written in simple language (Annex 14). To facilitate communication with children of varying abilities, an assent document with pictograms has been developed (Annex 15). All participants and their guardians will receive a comprehensive explanation of the project’s objectives, potential risks and benefits, methodologies, expected outcomes, and data usage policies. These explanations will be delivered in clear, simple language to ensure full comprehension. Furthermore, a commitment to transparent, community-centered, and timely communication will be upheld throughout the research process to maintain respect for all participants. Both parents and children will be explicitly informed of their right to withdraw from the study at any time without any negative consequences.

Data collection will occur in a comfortable and respectful environment, prioritizing the sensitivity of the children and their families. Where possible, sessions will be conducted in the participant’s home to minimize distress. Caregivers will be empowered to specify conditions necessary for their child’s comfort and well-being, and our team will accommodate these preferences. All data will be collected by personnel trained to work with neurodivergent children. Respect for participants will be upheld through the use of appropriate language, strictly avoiding derogatory, stigmatizing, discriminatory, obsolete, or incorrect terminology, following established best-practice guidelines (63). It is important to emphasize that the data collection methods are non-invasive and pose no risk of harm or injury to participants.

Participants will not receive financial compensation for their involvement. In alignment with current autism research guidelines, we recognize that caregivers value access to and understanding of research findings; the unavailability of results often leads to frustration (64). To address this, we will disseminate the study results through parent meetings, presenting the findings in an accessible manner. Additionally, we will offer individualized feedback on each child’s nutritional status along with personalized dietary recommendations to the caregivers as a form of compensation for their time and effort.

We acknowledge that parents of newly diagnosed autistic children may experience significant emotional distress, including intense sorrow and grief, due to the profound impact on their lives (65). A recent study also indicated that a high percentage of caregivers of autistic children in the province of Chimborazo suffer from depression (66). Accordingly, the research team will be trained to be mindful of these challenges, practicing active listening during interactions, providing comfort, and maintaining realistic expectations about the study’s scope and limitations.

Questionnaire data will be collected and managed using the Research Electronic Data Capture (REDCap) application. All information, including survey responses, collected data, and biological samples, will be de-identified and assigned a unique code to ensure confidentiality. Only the principal investigator and authorized researchers of “Proyecto Wiñay” will have access to the identified documentation. In any publications resulting from the project, participant confidentiality will be strictly maintained. The biological samples will be used exclusively for this study. They will be stored anonymously in our laboratory for the study’s duration and safely incinerated thereafter.

## 4 Discussion

The present is the protocol of the “Proyecto Wiñay”, a comparative cross-sectional study that will be conducted in Ecuador with the financial support of the Escuela Superior Politécnica de Chimborazo. The study aims to investigate relationships between objectively measured SMD, dietary patterns, nutritional status, and GM composition and diversity in autistic children and adolescents versus NA peers.

### 4.1 “Proyecto Wiñay” in the current Ecuadorian context

Feeding challenges in autistic individuals pose significant health concerns, particularly due to their impact on nutritional status (67, 68). SMD constitutes a key contributor to these challenges, yet its association with dietary patterns and GM characteristics remains unexplored in the Ecuadorian pediatric population. Our study aims to fill this gap by conducting an integrated assessment of SMD, dietary patterns, and GM characteristics in autistic children from the Andes, a group known to face high malnutrition rates and limited healthcare access (24). While other studies acknowledge the link between SMD and malnutrition in autism, our approach is distinct in its specific adaptation of methodologies to the Ecuadorian context. We hypothesize that SMD contributes to restrictive diets that subsequently alter GM composition and diversity, potentially exacerbating core autistic symptoms and perpetuating a cycle of malnutrition.

This research employs innovative techniques, including neurometrics to objectively assess SMD through measures of sensory gating and habituation as well as general motor behaviors. Additionally, GM characteristics will be evaluated in a population with high prevalence of intestinal parasitosis, a factor often overlooked in studies from high-income countries (69). Further, we will use WHO-standardized anthropometric measurements to assess three groups: autistic children, NA children, and children with developmental disorders other than autism. This comparative approach will provide valuable insights into the unique nutritional challenges and GM alterations specifically associated with autism.

Unlike previous Ecuadorian studies (e.g., Zurita et al., 2020), which focused primarily on nutritional status and GM, “Proyecto Wiñay” offers a more comprehensive approach by incorporating objective SMD analysis (70). Furthermore, our study will recruit a larger sample size from a more ethnically diverse population (including participants from both Quito and Riobamba cities) and across a broader age range. Although cross-sectional, our study has the potential to establish the basis for future longitudinal studies tracking GM changes or symptom severity linked to sensorimotor-mediated dietary patterns.

### 4.2 Study limitations

This study’s primary methodological limitation is its cross-sectional design, which inherently precludes the inference of causal relationships between exposures and outcomes. Our findings will thus be restricted to identifying non-causal associations that warrant future longitudinal or experimental validation. To enhance the robustness of our findings within this observational framework, we will employ multivariate regression models to control for key potential confounders (e.g., sex, age, socioeconomic status, use of medication). We will also conduct sensitivity analyses to assess the stability of these associations under different modelling assumptions.

The generalizability (external validity) of our findings will be constrained by the recruitment strategy. Sourcing participants from specialized private healthcare centers will likely yield a sample overrepresenting families of higher socioeconomic status and better healthcare access. This strategy may also bias the cohort toward more clinically severe ASD presentations, potentially underrepresenting children with milder phenotypes or those from marginalized communities who face barriers to diagnosis.

This sampling approach was chosen to safeguard internal validity and feasibility. Centralized recruitment was essential to secure a sufficient number of professionally diagnosed participants and to administer the study’s protocols consistently. Therefore, while the results will be most directly applicable to urban-dwelling autistic children within Ecuador’s private healthcare system, they will provide high-fidelity data on the hypothesized relationships under investigation.

This trade-off is justified for the hypothesis-generating aims of this study. Following Rothman’s principles, the initial test of a mechanistic pathway (e.g., between SMD and dietary patterns) requires a well-defined cohort where exposures and outcomes can be measured with precision, even at the expense of representativeness (71). The associations identified here will provide the foundational evidence necessary to design future longitudinal and population-representative studies.

Although the selected instruments have been validated in Spanish-speaking populations, their performance remains unverified within the specific sociocultural context of Ecuador. Despite linguistic proximity, cultural differences in symptom perception, expression, and reporting could potentially affect metric reliability and validity in our target population. To ensure conceptual and linguistic equivalence, we will evaluate the instruments’ cultural appropriateness and local applicability during a pilot phase, with findings informing necessary adjustments to the protocols.

For the present study, we will use a proprietary smartphone-based application that enables standardized, non-invasive neurometric assessment (21). This innovative approach offers a significant advantage over caregiver-reported SMD assessments. We acknowledge, however, that while this innovation addresses some limitations, it introduces challenges for future scalability and equity. The application’s exclusivity to iOS and its proprietary commercial model may constrain its widespread adoption in public health or clinical settings across Ecuador and other middle/low income countries, where resource limitations are prevalent. Consequently, while this tool enables objective data collection for our primary aims, a key secondary outcome of our investigation will be a critical assessment of its practical feasibility and implementation barriers.

The 24HDR method was selected for this study despite its well-documented limitations, such as recall and portion size estimation bias, due to its well-established feasibility, lower respondent burden, and strong validation profile (72, 73). Its utility as a standard is evidenced by its use as the reference method in nearly 75% of validation studies for other dietary assessment tools, such as food frequency questionnaires (74).

Self-medication represents an important limitation that our study will address. In Ecuador, self-medication, particularly with antibiotics, is common across various demographic groups (75). Excluding participants based on reported medication use would severely constrain recruitment. We will therefore account for this potential confounder by adjusting for medication use in multivariable statistical models when examining associations between gut microbiota composition and other outcomes. Specifically, medication use will be included as a covariate in linear models for alpha-diversity analyses and in PERMANOVA for beta-diversity comparisons. Finally, following previously used approaches (76), we will evaluate the robustness of our findings through sensitivity analyses that leverage the detailed medication data collected, exploring drug dosage, the specific reason for medication, and the prescribing source.

Another potential limitation of our study is that no formal cognitive ability assessment will be conducted. Cognitive level can influence dietary patterns independently of sensorimotor differences, for example, by affecting a child’s ability to express food preferences or aversions. Moreover, 30–40% of children with ASD also have co-occurring intellectual disability, a rate substantially higher than among NA peers (77). To address this potential confounding, we will use CARS scores as a proxy for phenotypic severity within the ASD group. Although CARS does not measure cognitive ability directly, it correlates with functional impairment and will allow us to explore whether associations differ by severity level. Nevertheless, residual confounding by unmeasured cognitive differences cannot be entirely ruled out.

The broad age range (3–17 years) of our sample represents another limitation, given the significant developmental heterogeneity within this period. While a narrower range would reduce variability, it would also compromise sample size and generalizability. To address this, age will be included as a covariate in all multivariate models to adjust for its potential confounding effects. Additionally, we have selected age-appropriate instruments to ensure that measurements are sensitive to developmental stages. Nevertheless, residual age-related variability may still influence our findings, and we acknowledge this trade-off.

### 4.3 Impact and future directions

This cross-sectional study is positioned to make several contributions to the field. By integrating objective neurometrics, detailed nutritional assessment, and gut microbiota analysis, “Proyecto Wiñay” will provide a foundational dataset on the SMD-nutrition-microbiota relationship in a previously understudied Andean population. The findings will directly address the critical limitation of relying on subjective, caregiver-reported SMD measures, offering a more robust, biologically-grounded understanding of how sensorimotor processing drives dietary patterns.

If our hypothesis is supported, the results will provide mechanistic insight into the cycle wherein SMD contributes to restrictive diets, nutritional imbalance, and gut dysbiosis, potentially exacerbating core autistic symptoms. This evidence is a necessary precursor to breaking this cycle. Specifically, identifying which sensorimotor profiles most strongly predict nutritional deficits could inform the development of sensorimotor-informed nutritional interventions. For instance, strategies could move beyond generic advice to include structured mealtime routines for those with heightened or reduced reactivity.

Furthermore, characterizing the gut microbiome in a population with a high endemic burden of intestinal parasitosis will challenge the Western-centric models that dominate the current literature. Data on how common pathogens interact with the autistic gut microbiome may reveal unique microbial adaptations or dysbiotic patterns, contributing to a more globally representative understanding of the gut-brain axis. This is crucial for developing public health and nutritional strategies that are relevant to middle/low income countries.

Finally, the evaluation of an accessible, smartphone-based neurometric tool addresses a significant barrier to research and care in resource-limited settings. Assessing its feasibility in this context provides critical preliminary data on its potential for future applications in remote screening, monitoring sensory phenotypes, and evaluating interventions, thereby helping to bridge the diagnostic and care gap between urban and rural areas.

## Glossary

24-HDR: 24-hour dietary recall
AQ-10: 10-item Autism Spectrum Quotient
ASD: Autism Spectrum Disorder
CARS: Childhood Autism Rating Scale
GM: gut microbiota
ICD-10: International Classification of Diseases, 10th Edition
NA: non-autistic
POSI: Parent’s Observations of Social Interactions
PPI: pre-pulse inhibition of the startle response
SH: startle habituation
SMD: sensorimotor integration differences
