# Supplementary material for: Sensorimotor integration, nutrition and gut microbiota in Ecuadorian autistic children – “Proyecto Wiñay”: a research protocol for a comparative cross-sectional study
Source: Front Psychiatry. 2026 Apr 30;17:1721567. doi: 10.3389/fpsyt.2026.1721567 (PMC13173513; doi:10.3389/fpsyt.2026.1721567)
Supplement: Supplementary file 1 [file DataSheet1.zip › Data Sheet 1.PDF]

Supplementary material

1. Supplementary Data

Annex 1: Survey about personal and sociodemographic data, administered to the caregivers by research personnel.

1.1. Encuesta dirigida al informante, a ser llenada por el investigador de campo

1.1.1. Fecha y hora

1.1.1.1. Fecha (dd/mm/aa):___________

1.1.1.2. Hora (24H00): _____________

1.1.2. Datos del niño/a

1.1.2.1. Código personal del niño/a (aaaammdd-nnnn):

____

1.1.2.2. Nombre del niño/a (Nombre #1 / Nombre #2 / Apellido #1 / Apellido #2):

____

1.1.2.3. Lugar de residencia del niño/a (provincia/cantón/parroquia/calles

____

1.2.1. Datos del niño/a

1.2.1.1. Fecha de nacimiento (dd/mm/aaaa):___________

1.2.1.2. Edad en años (nn):_________

1.2.1.3. Sexo biológico al nacer: Masculino ( ), Femenino ( )

1.2.1.4. Número de años de educación (formal) recibida por el niño/a:_________

1.2.1.5. Nivel de educación que el niño/a esta cursando actualmente: Educación inicial ( ), Educación General Básica ( ), Bachillerato ( ), No asiste a la escuela ( )

1.2.1.6. En caso de asistir a la escuela, indicar el grado o nivel que está cursando: ___________________________________

1.2.1.7. En caso de no asistir a la escuela indicar la razón

____

1.1.3. Datos del informante

1.1.3.1. Nombre del informante (Nombre #1 / Nombre #2 / Apellido #1 / Apellido #2):

____

1.1.3.2. Relación de parentesco con el niño/a: Padre ( ), Madre ( ), Otro – especifique ( )

____

1.1.3.3. Número telefónico del informante:

____

1.1.3.4. Correo electrónico del informante:

____

1.2. Encuesta dirigida al informante, a ser llenada por el investigador de campo

1. Fecha y hora
   1.1. Fecha (dd/mm/aa): ___________
   1.2. Hora (24H00): _____________

2. Datos del niño/a
   2.1. Código personal del niño/a (aaaammdd-nnnn):

____

3. Datos del padre y/o la madre

3.1. Edad del padre (años):

____

3.2. Número de años de educación (formal) recibida del padre:

____

3.3. Edad de la madre (años):

____

3.4. Número de años de educación (formal) recibida de la madre:

____

Annex 2. Survey on the socioeconomic status of the child/adolescent's household, addressed to the informant (preferably parents or primary caregivers). Can be self-administered.

NOTA: Este cuestionario está dirigido a la persona informante, que de preferencia será la madre, padre o cuidador/a principal de la niña o del niño. <u>Puede ser auto-administrado.</u>

Marque con una X en cada pregunta, lo que corresponda a su situación:

A. *CARACTERÍSTICAS DE LA VIVIENDA*

a ¿Cuál es el tipo de vivienda donde vive?

Suite de lujo ☐ Cuarto(s) en casa de inquilinato ☐

Departamento en casa o edificio ☐ Casa/Villa ☐

Mediagua ☐ Rancho ☐

Choza/ Covacha/Otro ☐

b El material predominante de las paredes exteriores de la vivienda es de:

Hormigón☐Ladrillo o bloque ☐ Adobe/ Tapia ☐

Caña revestida o bahareque/ Madera ☐

Caña no revestida/ Otros materiales ☐

c El material predominante del piso de la vivienda es de:

Duela, parquet, tablón o piso flotante ☐

Cerámica, baldosa, vinil o marmetón ☐

Ladrillo o cemento ☐ Tabla sin tratar ☐

Tierra/ Caña/ Otros materiales ☐

d ¿Cuántos cuartos de baño con ducha de uso exclusivo tiene este hogar?

☐ No tiene cuarto de baño exclusivo con ducha en el hogar

☐ Tiene 1 cuarto de baño exclusivo con ducha

☐ Tiene 2 cuartos de baño exclusivos con ducha

☐ Tiene 3 o más cuartos de baño exclusivos con ducha

e El tipo de servicio higiénico con que cuenta este hogar es:

No tiene ☐ Letrina ☐ Con descarga directa al mar, río, lago o quebrada ☐

Conectado a pozo ciego ☐ Conectado a pozo séptico ☐
Conectado a red pública de alcantarillado ☐

B. *ACCESO A TECNOLOGÍA*

a ¿Tiene este hogar servicio de internet?

Si ☐ No ☐

b ¿Tiene computadora de escritorio?

Si ☐ No ☐

c ¿Tiene computadora portátil?

Si ☐ No ☐

d ¿Cuántos celulares activados tienen en este hogar?

No tiene celular nadie en el hogar ☐

Tiene 1 celular ☐

Tiene 2 celulares ☐

Tiene 3 celulares ☐

Tiene 4 ó más celulares ☐

e ¿Tiene este hogar servicio de teléfono convencional?

Si ☐ No ☐

f ¿Tiene cocina con horno?

Si ☐ No ☐

g ¿Tiene refrigeradora?

Si ☐ No ☐

h ¿Tiene lavadora?

Si ☐ No ☐

i ¿Tiene equipo de sonido?

Si ☐ No ☐

j ¿Cuántos TV a color tienen en este hogar?

No tiene TV a color en el hogar ☐
Tiene 1 TV a color ☐

Tiene 2 TV a color ☐
Tiene 3 ó más TV a color ☐

k ***¿Cuántos vehículos de uso exclusivo tiene este hogar?***

No tiene vehículo exclusivo para el hogar ☐
Tiene 1 vehículo exclusivo ☐

Tiene 2 vehículos exclusivos ☐
Tiene 3 ó más vehículos exclusivos ☐

l ***¿Alguien en el hogar compra vestimenta en centros comerciales?***

Si ☐ No ☐

m ***¿En el hogar alguien ha usado internet en los últimos 6 meses?***

Si ☐ No ☐

n ***¿En el hogar alguien utiliza correo electrónico que no es del trabajo?***

Si ☐ No ☐

o ***¿En el hogar alguien está registrado en una red social?***

Si ☐ No ☐

p ***Exceptuando los libros de texto o manuales de estudio y lecturas de trabajo. ¿Alguien del hogar ha leído algún libro completo en los últimos 3 meses?***

Si ☐ No ☐

q ***¿Cuál es el nivel de instrucción del jefe del hogar?***

Sin estudios ☐ Primaria incompleta ☐ Primaria completa ☐

Secundaria incompleta ☐ Secundaria completa ☐

Hasta 3 años de educación superior ☐
4 ó más años de educación superior (sin postgrado) ☐ Postgrado ☐

r ***¿Alguien en el hogar está afiliado o cubierto por el seguro del IESS (general, voluntario o campesino) y/o seguro del ISSFA o ISSPOL?***

Si ☐ No ☐

s ***¿Alguien en el hogar tiene seguro de salud privada con hospitalización, seguro de salud privada sin hospitalización, seguro internacional, seguros municipales y de Consejos Provinciales y/o seguro de vida?***

Si ☐ No ☐

t ***¿Cuál es la ocupación del jefe o jefa del hogar? Escriba y luego seleccione el más adecuado***

Personal directivo de la Administración Pública y de empresas ☐

☐

Profesionales científicos e intelectuales

Técnicos y profesionales de nivel medio ☐

Empleados de oficina ☐

Trabajador de los servicios y comerciantes ☐

Trabajadores calificados agropecuarios y pesqueros ☐

Oficiales operarios y artesanos ☐

Operadores de instalaciones y máquinas ☐

Trabajadores no calificados ☐

Fuerzas Armadas ☐ Desocupados ☐ Inactivos ☐

- ***Tiene agua potable***

Si ☐ No ☐

Annex 3. Personal Medical History Questionnaire, addressed to the informant (parents or primary caregivers). Administered by the research personnel.

Encuesta dirigida al informante, a ser llenada por el investigador de campo

3.1. Fecha y hora
3.1.1. Fecha (dd/mm/aa): ___________
3.1.2. Hora (24H00): _____________

3.2. Datos del niño/a
3.2.1. Código personal del niño/a (aaaammdd-nnnn):

3.3. Cuestionario médico
3.3.1. Durante el ÚLTIMO MES, ¿El niño/a ha tomado alguna de las siguientes medicinas? (Encuestador, explicar en qué consiste cada uno. Por ejemplo, antibiótico: medicina contra las bacterias):
Antibiótico ( ), Antiviral ( ), Antiparasitario ( ), Antimicótico ( ), Ninguna ( ).

3.3.2. En caso de haber recibido un medicamento de los citados en la última pregunta, llene la siguiente información con ayuda del informante

Medicamento	Dosis en 24 horas	Motivo por el cual consumió el medicamento	Persona quien indicó tomar el medicamento

3.3.3. ¿Tiene el niño/a algún trastorno del neurodesarrollo diagnosticado? Sí ( )No ( ).

3.3.4. En caso de responder afirmativamente en la pregunta anterior, seleccione que trastorno padece:

____

frontiers

____
____
____
____

3.3.5. ¿Tiene el niño/a algún déficit visual? Si ( ) No ( ).

3.3.6. En caso de responder afirmativamente en la pregunta anterior, ¿Dispone el niño/a de lentes para suplir este déficit? Sí ( ) No ( )

3.3.7. ¿Tiene el niño/a algún déficit auditivo? Si ( ) No ( ).

3.3.8. En caso de responder afirmativamente en la pregunta anterior, ¿Dispone el niño/a de audífonos para suplir este déficit? Sí ( ) No ( ).

3.3.9. ¿Tiene el niño/a algún familiar con alguna enfermedad neuropsiquiátrica diagnosticada? (Encuestador, restringir la pregunta a primer y segundo grado de consanguinidad, explicar que familiares abarcan estos grados). Sí ( ) No ( )

3.3.10. En caso afirmativo en la pregunta anterior, ¿Qué enfermedad?

____

3.3.11. En caso de responder afirmativamente a la pregunta anterior, indicar qué grado de parentesco tiene el niño/a en relación con la persona afectada

____

3.3.12. ¿Cuál fue la edad de la madre al gestar al niño/a?

____

3.3.13. ¿Cuál fue la edad del padre al gestar al niño/a?

____

3.3.14. ¿Toma el niño/a medicación de manera habitual (Encuestador, la definición de habitual es más de tres veces por semana)? Si ( ), No ( ).

3.3.15. En caso de responder afirmativamente a la pregunta anterior, indique los datos de cada medicamento.

Medicamento	Motivo por el cual consumió el medicamento	Mejoría sintomática en relación a la causa por la que consume el medicamento. (SI o NO)	Persona quien indicó tomar el medicamento

3.3.16. ¿Toma el niño/a medicación natural/tradicional de manera habitual (Encuestador, la definición de habitual es más de tres veces por semana.)? Si ( ), No ( ).

3.3.17. En caso de responder afirmativamente a la pregunta anterior, indique los datos de cada medicamento.

Medicamento	Motivo por el cual consumió el medicamento	Mejoría sintomática en relación a la causa por la que consume el medicamento. (SI o NO)	Persona quien indicó tomar el medicamento

3.3.18. ¿Ha presentado el niño/a síntomas gastrointestinales (Encuestador, explicar en qué consisten los síntomas gastrointestinales) durante los últimos TRES MESES? Si ( ) No ( )

3.3.19. En caso afirmativo en la última pregunta, por favor indique qué síntomas padeció el niño/a:

____

3.3.20. ¿Tiene el niño/a el diagnóstico de alguna o varias de las siguientes enfermedades?: Rinitis alérgica ( ). Asma bronquial ( ). Dermatitis atópica ( ). Alergia alimentaria ( ). Alergia a la picadura de insectos ( ). Conjuntivitis alérgica ( ). Ninguna ( )

3.3.21. ¿Al niño/a se le ha diagnosticado Trastorno del Espectro Autista (TEA)? Sí ( ) No ( ). En caso de que haya respondido NO, puede terminar aquí el cuestionario.

3.3.22. Indique el año en que se realizó el diagnóstico (aaaa):____________________

3.3.23. Durante los últimos TRES MESES, ¿Ha implementado cambios en el régimen dietético de su niña o niño con fines terapéuticos, o sintomáticos? Sí ( ) No ( )

3.3.24. En caso afirmativo, por favor indique brevemente qué tipo de dieta ha seguido

3.3.25. En caso afirmativo en la pregunta anterior, ¿Cuáles han sido los resultados de esta dieta? Mejora sintomática ( ), Empeoramiento sintomático ( ), Se mantuvo igual, ningún efecto palpable ( )

3.3.26. El diagnóstico de TEA, ¿Se acompañó con una prueba genética? Sí ( )No ( )

3.3.27. En caso afirmativo, por favor indique cuál fue el resultado de este examen

____

3.3.28. El diagnóstico de TEA, ¿Se acompañó con una prueba metabólica? Sí ( ) No ( )

3.3.29. En caso afirmativo, por favor indique cuál fue el resultado de este examen

____

3.3.30. ¿En los últimos TRES MESES, su niña o niño ha recibido psicoterapia? Sí ( ) No ( )

3.3.31. En caso afirmativo, por favor indique qué cantidad de sesiones ha recibido en los últimos TRES MESES

____

Annex 4: Data about dietary patterns

FECHA: dd/mm/aaaa _____/____/______________ ***Hora:***___:______

1. **RECORDATORIO DE 24 HORAS:**

NOTA: Este cuestionario está dirigido a la persona informante, que de preferencia será la madre, padre o cuidador/a principal de la niña o del niño. A llenarse por el personal investigador. Utilizar el ÁLBUM FOTOGRÁFICO DE LAS PORCIONES ALIMENTARIAS EN ECUADOR DE HERRERA ET AL..

A continuación, se le pedirá que recuerde todo lo que su hija/o comió y bebió durante tres días: dos días entre semana y un día en fin de semana. Por favor, sea lo más detallado posible. Incluya:

- **Hora:** A qué hora se consumió cada alimento o bebida.
- **Tiempo de comida:** comidas principales (desayuno, almuerzo, cena) y colaciones.
- **Tipo de alimento:** Nombre específico del alimento o bebida.
- **Cantidad:** Utilice medidas caseras (por ejemplo, taza, cucharada) o estimaciones (por ejemplo, la mitad de un plato). Primero el plato inicial y luego el plato final.
- **Lugar:** Dónde se consumió (en casa, en la escuela, en un restaurante).

DIA 1 ENTRE SEMANA; FECHA: ___________________________

Horario	Tiempo de comida	Alimentos y bebidas consumidos	Cantidad	Lugar de consumo	Notas adicionales	Intercambios
	Desayuno					
	Colación matutina					
	Almuerzo					

	Colación vespertina					
	Cena					
	Colación nocturna					

DÍA 2: DÍA ENTRE SEMANA; FECHA: ________________________

Horario	Tiempo de comida	Alimentos y bebidas consumidos	Cantidad	Lugar de consumo	Notas adicionales	Intercambios
	Desayuno					
	Colación matutina					
	Almuerzo					
	Colación vespertina					

	Cena					
	Colación nocturna					

DÍA 3: DíA DE FIN DE SEMANA; FECHA: ____________________

**Horario**	**Tiempo de comida**	**Alimentos y bebidas consumidos**	**Cantidad**	**Lugar de consumo**	**Notas adicionales**	**Intercambios**
	Desayuno					
	Colación matutina					
	Almuerzo					
	Colación vespertina					
	Cena					
	Colación nocturna					

Annex 5: Data about nutritional status

ESTADO NUTRICIONAL.

a. Datos antropométricos

Nota: Tomar los valores antropométricos 3 veces con un lapso de 5 minutos entre cada medición. Tomar las mediciones dos horas después de haber comido. La evaluación deberá hacer la investigadora de campo.

Peso 1 en kg: _____ Peso 2 en kg:____ Peso 3 en kg:______
Talla 1. en cm: _____ Talla 2. en cm: _____ Talla 3. en c : _____
Circunferencia abdominal 1 en cm: _____ Circunferencia abdominal 2 en cm: _____
Circunferencia abdominal 3 en cm _____
Circunferencia de cuello 1 en cm:____ Circunferencia de cuello 2 en cm:_____
Circunferencia de cuello 3 en cm:____

b. Bioimpedancia

Nota: Tomar las mediciones dos horas después de haber comido, después de haber orinado. Guardar el .pdf con el código correspondiente para luego pasar a la base datos.
Se hizo la medición: si ( ), no ( )
Observación:____

Annex 6: Semi-structured interview guide for parents or primary caregivers (6.1) and for health professionals (6.2) to be used by research staff.

6.1. GUÍA DE ENTREVISTA SEMIESTRUCTURADA DIRIGIDA A PADRES, MADRES O CUIDADORAS. A REALIZAR POR EL PERSONAL INVESTIGADOR.

Se realiza esta investigación para comprender mejor las experiencias y desafíos que enfrentan los padres de niños con autismo en relación con la alimentación. Su participación en este estudio es voluntaria, sin embargo es muy importante porque contribuirá a mejorar el conocimiento sobre las necesidades de las familias de las niñas/os con autismo y a diseñar intervenciones adecuadas a la realidad de las niñas/os y familias que viven en el Ecuador. Además, tendrá la oportunidad de compartir sus experiencias con otros padres y recibir apoyo en caso de ser necesario. Es posible que al compartir sus experiencias pueda experimentar algunas emociones como nostalgia o frustración. Si esto ocurre, el moderador estará presente para brindarle apoyo.

Toda la información que usted proporcione será tratada de manera confidencial. Sus respuestas serán anónimas y no se asociará con su nombre en ningún informe o publicación. Usted puede decidir dejar de participar en el estudio en cualquier momento sin dar ninguna explicación. Esto no afectará de ninguna manera a la atención que recibe su hija/o.

La entrevista tendrá una duración de unos 30-40 minutos aproximadamente, en caso de que usted no pueda realizarla de manera personal, podemos organizar una reunión virtual por zoom u otra plataforma que permita realizar la entrevista. Antes de realizar la entrevista es necesario contarle de qué se trata el proyecto y en caso de que usted acepte participar deberá firmar el consentimiento informado. Una vez que firme este documento procederemos con la entrevista.

Se realizará el siguiente procedimiento:
1. Se le enviará el enlace de Zoom al momento de confirmar la fecha y hora, junto con el consentimiento informado, previo a la entrevista con el participante. En caso de que la entrevista sea de manera personal se coordinará lugar, día y hora.
2. Durante la reunión se ofrece un cordial saludo al participante, agradeciéndole por su participación y por dedicar un espacio de su tiempo.
3. Controlar el tiempo de la entrevista para lograr despedirse y reiterar el agradecimiento por su colaboración en el estudio.
4. Se procederá a explicar al participante que se grabará la entrevista, omitiendo su información personal con el fin de poder utilizar sus respuestas en la investigación con fines académicos.
5. Evite inducir respuestas, es importante que el entrevistado se exprese libremente sobre la pregunta sin desviarse del tema, si algo no queda claro se replantea la pregunta.

1. Buenos días. tardes… Para empezar la entrevista quisiera agradecerle por su colaboración, me gustaría preguntarle algunos datos, vamos a comenzar por su nombre, luego me gustaría que me indique su edad, qué relación tiene usted con la hija/o, es decir, usted es su madre.. padre.. abuelita… (etc, lo que indique el/la participante) Por favor indíqueme en qué ciudad o lugar vive? ¿es urbana o rural? ¿cuántas hijas/hijos tiene? ¿Cuál es su estado civil? ¿En qué trabaja? Anotar relación

con la participante: ____________________________ EDAD:_____

Para participar en la entrevista debe tener firmado el consentimiento informado.

2. ¿Cómo fueron los primeros años de alimentación de su hija/o? ¿Hubo alguna dificultad o evento significativo relacionado con la alimentación (por ejemplo, dificultades para mamar, rechazo a papillas)?

3. ¿Recibió su hijo lactancia materna exclusiva?

4. ¿Cómo fue la transición de la leche materna a la alimentación complementaria? ¿Utilizó algún método de alimentación inicial? ¿Ha escuchado sobre BLW o dieta mixta?

5. ¿Cómo fue el proceso de introducción de nuevos alimentos? ¿Hubo alguna resistencia o preferencia por ciertas texturas o sabores?

6. ¿Cómo describiría el desarrollo general de su hija/o en relación a sus pares?

7. ¿Usted cree que la alimentación ha afectado el crecimiento o desarrollo de su hija/o?

4. ¿Puede describir un día típico de alimentación de su hija/o? ¿Incluye horarios regulares para las comidas y colaciones?

5. ¿Cuáles son los alimentos favoritos y menos favoritos de su hija/o? ¿Existen alimentos que rechaza por completo?

-

6. ¿Hay alguna textura o sabor en particular que le provoque aversión (rechazo, asco)? ¿Cómo reacciona a alimentos con diferentes temperaturas (frío, caliente) su hija/o?

7. ¿Qué tipo de comportamientos observa durante las comidas? (ej., jugar con la comida, rechazar bocados, autoalimentarse, etc.)

8. ¿Cómo es el ambiente en el que su hija/o come? ¿Hay algún ritual o rutina específica antes, durante o después de las comidas?

9. ¿Cree que la alimentación influye en su capacidad de concentración, aprendizaje o habilidades sociales de su hija/o?

10. ¿Ha notado algún problema de salud relacionado con la alimentación de su hija/o? (ej., estreñimiento, bajo peso, reflujo, alergias alimentarias)

11. ¿Cómo afectan emocionalmente los problemas con los alimentos a su hija/o? ¿Ha notado cambios en su comportamiento, como mayor aislamiento o irritabilidad

12. ¿Cómo ha influido la situación alimentaria de su hija/o en el nivel de estrés de la familia?

13. ¿Qué estrategias han implementado para hacer que las comidas sean más agradables y menos estresantes para toda la familia?

14. ¿Utiliza algún tipo de suplemento nutricional? Si la respuesta es sí, indique la razón, cuál suplemento utiliza y quién se lo recomendó.

15. ¿Sigue alguna dieta especial (ej., sin gluten, sin caseína)? ¿Por qué?

16. ¿Cuenta con algún tipo de apoyo (familiar, amigos, profesionales) para abordar los problemas con los alimentos de su hija/o?

17. ¿Cuáles son sus principales objetivos en cuanto a la alimentación de su hija/o?

18. ¿Qué tipo de apoyo adicional necesitaría para mejorar la alimentación de su hija/o?

19. ¿Qué aspectos de la alimentación de su hija/o le resultan más fáciles de manejar y cuáles representan un mayor desafío?

20. ¿Existen ciertos alimentos o situaciones relacionadas con la comida que su hija/o disfruta especialmente y que facilitan la alimentación?

21. ¿Considera que el presupuesto familiar influye en la variedad de alimentos que puede ofrecerle a su hija/o?

22. Existen alimentos que le gustaría incluir en la dieta de su hija/o pero que, por motivos económicos, no puede hacerlo?

Observaciones:
____

____

6.2. GUÍA DE ENTREVISTA SEMIESTRUCTURADA PERSONAL DE SALUD

En esta investigación se pretende conocer las experiencias y desafíos que enfrenta el personal de salud que atiende a niñas/os con autismo en relación. Su participación en este estudio es voluntaria, sin embargo es muy importante porque contribuirá a identificar situaciones que experimenta el personal de salud al atender a niñas/os y familias de niñas/os con autismo en el Ecuador.

Toda la información que usted proporcione será tratada de manera confidencial. Sus respuestas serán anónimas y no se asociará con su nombre en ningún informe o publicación. Usted puede decidir dejar de participar en el estudio en cualquier momento sin dar ninguna explicación. Esto no le afectará de ninguna manera.

La entrevista tendrá una duración de unos 30-40 minutos aproximadamente, en caso de que usted no pueda realizarla de manera personal, podemos organizar una reunión virtual por zoom u otra plataforma que permita realizar la entrevista. Antes de realizar la entrevista es necesario contarle de qué se trata el proyecto y en caso de que usted acepte participar deberá firmar el consentimiento informado. Una vez que firme este documento procederemos con la entrevista.

Se realizará el siguiente procedimiento:

1. Se le enviará el enlace de Zoom o se concertará una reunión al momento de confirmar la fecha y hora, junto con el consentimiento informado, previo a la entrevista con el participante. En caso de que la entrevista sea de manera personal se coordinará lugar, día y hora.
2. Durante la reunión se ofrece un cordial saludo al participante, agradeciéndole por su participación y por dedicar un espacio de su tiempo.
3. Controlar el tiempo de la entrevista para lograr despedirse y reiterar el agradecimiento por su colaboración en el estudio.
4. Se procederá a explicar al participante que se grabará la entrevista, omitiendo su información personal con el fin de poder utilizar sus respuestas en la investigación con fines académicos.
5. Evite inducir respuestas, es importante que el entrevistado se exprese libremente sobre la pregunta sin desviarse del tema, si algo no queda claro se replantea la pregunta.

Una vez que ha firmado el consentimiento informado, para empezar la entrevista me gustaría preguntarle algunos datos, vamos a comenzar por su nombre…

Datos Generales del Entrevistado:

Nombre:	Área de Especialización:
Profesión:	Años de trabajo en esa área:
Edad	Sexo

Años de Experiencia:	Institución donde labora:

1. ¿Podría describir brevemente su experiencia trabajando con niñas/os y adolescentes con autismo?

2. ¿Cuáles son los principales desafíos que identifica al establecer un primer contacto con una niña/o, o adolescente con autismo y su familia?

3. ¿Qué dificultades encuentra en la comunicación con niñas/os y adolescentes con autismo?

4. ¿Existen herramientas o estrategias específicas que utilice para facilitar la comunicación?

5. ¿Qué comportamientos o conductas suelen presentar las niñas/os y adolescentes con autismo que dificultan la atención médica?

   ¿Cómo aborda estas situaciones?

6. ¿Qué desafíos encuentra al administrar medicamentos a niñas/os y adolescentes con autismo?

   ¿Existen resistencias o dificultades particulares?

7. ¿Observa alguna dificultad específica en relación con la alimentación y nutrición de estos pacientes?

8. ¿Existen necesidades nutricionales particulares que deban ser consideradas?

9. ¿Considera que las niñas/os con Autismo deberían consumir suplementos nutricionales?

   Detalle cuáles serían:

10. ¿Qué obstáculos encuentra para garantizar una buena alimentación a estos pacientes?

11. ¿Qué tipo de colaboración considera esencial para brindar una atención integral a estos pacientes?

12. ¿Con qué otros profesionales suele trabajar?

13. ¿Considera que la formación que recibió le proporciona las herramientas necesarias para atender a niñas/os y adolescentes con autismo?

14. ¿Qué aspectos considera que deberían reforzarse en la formación de los profesionales de la salud?
15. ¿Cuáles considera que son los principales recursos que faltan para brindar una atención de calidad a estos pacientes? (personal especializado, materiales, infraestructuras etc.)

16. ¿Cuáles son las principales expectativas de las familias de niñas/os y adolescentes con autismo en relación con la atención médica?

    ¿Cómo gestiona estas expectativas? ¿Cómo ha sido su experiencia con los padres/madres/cuidadores de las niñas/os y adolescentes?

17. ¿Qué barreras burocráticas o administrativas considera que dificultan la atención a estos pacientes?

18. ¿Qué aspectos positivos destaca en la atención a niñas/os y adolescentes con autismo en su institución?

19. ¿Qué fortalezas ha identificado en su propio trabajo?

20. ¿Qué recomendaciones daría para mejorar la atención a niñas/os y adolescentes con autismo en el sistema de salud?

21. ¿Podría compartir alguna experiencia positiva en su trabajo con estos pacientes?

22. ¿Qué le motiva a seguir trabajando en esta área?

"Muchas gracias por dedicar su tiempo a esta entrevista. Su experiencia y conocimientos son fundamentales para comprender mejor los desafíos y oportunidades en la atención a niñas/os y adolescentes con autismo”

frontiers

Annex 7: Parent's Observations of Social Interactions (POSI)

ENCUESTA DE OBSERVACIÓN DE INTERACCIÓN SOCIAL (POSI).

A continuación se solicita que marque **CON UNA X SOLAMENTE UNA DE LAS OPCIONES**, la que crea más adecuada para dar respuesta a cada una de las preguntas. **DEJE LA ÚLTIMA COLUMNA VACÍA**. Recuerde **NO EXISTEN RESPUESTAS EQUIVOCADAS.**

		Siempre	Usualmente	A veces	Rara vez	Nunca	Puntuación
1	¿Su hijo/a se interesa por jugar con otros niños?						
2	¿Su hijo/a señala con el dedo para mostrar algo que le interesa?						
3	¿Su hijo/a le trae objetos para compartir su interés con usted?						
4	¿Su hijo/a responde cuando usted le llama por su nombre?						
5	¿Su hijo/a imita lo que usted hace (por ejemplo, saludar, hacer gestos, movimientos)?						
6	¿Su hijo/a le mira a los ojos cuando le habla o juega con él/ella?						
7	¿Su hijo/a entiende lo que usted le dice sin necesidad de gestos?						

Annex 8: Autism Quotient-10 items (AQ-10) instrument (child version) aimed at the informant (preferably parents or primary caregivers). Self-administered.

ENCUESTA HABILIDADES SOCIALES (AQ-10 versión infantil)

NOTA: *este cuestionario será llenado por el padre, madre o cuidadora principal de la/el niña/o.*

FECHA: dd/mm/aaaa _____/____/_ ***Hora:***___:_

A continuación se solicita que marque **CON UNA X SOLAMENTE UNA DE LAS OPCIONES**, la que crea más adecuada para dar respuesta a cada una de las preguntas. **DEJE LA ÚLTIMA COLUMNA VACÍA**. Recuerde **NO EXISTEN RESPUESTAS EQUIVOCADAS.**

		Totalmente de acuerdo	Parcialmente de acuerdo	Parcialmente en desacuerdo	Totalmente en desacuerdo
1	La niña/niño con frecuencia escucha sonidos que otros no escuchan.				
2	La niña/niño tiende a ver todo, en lugar de concentrarse en los pequeños detalles.				
3	Estando en grupo, la niña/niño fácilmente puede seguir las conversaciones de personas diferentes.				
4	A la niña/niño le resulta fácil pasar de una actividad a otra.				
5	La niña/niño no sabe cómo mantener una conversación con su / sus compañeros.				
6	La niña/niño				

	es buena/bueno socializando y conversando.				
7	A la niña/niño le resulta difícil entender las intenciones o sentimientos de los personajes cuando se le lee un cuento.				
8	Cuando la niña/niño estuvo en preescolar, disfrutó jugar con otras/otros niñas/niñas interpretando personajes o asumiendo roles.				
9	A la niña/niño le resulta fácil entender lo que alguien está pensando o sintiendo con sólo mirar su cara.				
10	A la niña/niño le resulta difícil hacer nuevos amigos.				

Annex 9: Autism Quotient-10 items (AQ-10) instrument (adolescent version) aimed at the informant (preferably parents or primary caregivers). Self-administered.

Cuestionario AQ10 versión adolescente (12–15 años)

Por favor marque una opción por cada afirmación:

Nº	Frase	Totalmente de acuerdo	Algo de acuerdo	Algo en desacuerdo	Totalmente en desacuerdo
1	Suele notar patrones en las cosas todo el tiempo				
2	Normalmente se concentra más en el panorama general que en los pequeños detalles				
3	En un grupo social, puede seguir fácilmente las conversaciones de varias personas				
4	Si hay una interrupción, recupera rápidamente lo que estaba haciendo				
5	Con frecuencia descubre que no sabe cómo mantener una conversación				
6	Es bueno en pequeños diálogos sociales ("chitchat")				
7	Cuando era más pequeño, le gustaba jugar a juegos de simulación con otros niños				
8	Le resulta difícil imaginar cómo sería ser otra persona				
9	Le resultan fáciles las situaciones sociales				
10	Le cuesta hacer nuevos amigos				

Puntuación:

- Se asigna 1 punto por "Totalmente de acuerdo" o "Algo de acuerdo" a las afirmaciones 1, 5, 8 y 10.
- Se asigna 1 punto por "Totalmente en desacuerdo" o "Algo en desacuerdo" a las afirmaciones 2, 3, 4, 6, 7 y 9.
- Si el puntaje es 6 o más, se recomienda considerar una evaluación especializada

Annex 10: Childhood Autism Rating Scale (CARS) instrument to be administered to the caregivers by research staff.

NOTA: Este cuestionario está dirigido a la persona informante, que de preferencia será la madre, padre o cuidador/a principal de la niña o del niño. A llenarse por el personal investigador.

ESCALA DE MEDICION DE AUTISMO EN LA NIÑEZ (C.A.R.S.)
Eric Schopler, Ph.D.,Robert J. Reichler, M.D., And Barbara Rochen Renner, Ph.D.

I. Relación con los demás:

1 1.5 2 2.5 3 3.5 4

Sin dificultades. Cierta timidez		*Evita la mirada. Exigente. Excesivamente tímido. Dependencia paterna excesiva*		*Actitud distante. Dificultades en la interacción*		*Muy distante. Muy poca interacción*

II. Imitación:

1 1.5 2 2.5 3 3.5 4

Sin dificultades		*Imita conductas simples (aplaudir/sonidos)*		*Imita algunas veces con gran esfuerzo del adulto*		*Raramente o nunca imita, ni con ayuda del adulto*

III. Afecto:

1 1.5 2 2.5 3 3.5 4

Sin dificultades		*En ocasiones, tipos y grados de respuesta emocional son inadecuados*		*Respuesta emocional inadecuada (por exceso o por defecto)*		*Respuesta raramente apropiada (rigidez afectiva)*

IV. Uso del cuerpo:

1	1.5	2	2.5	3	3.5	4
Se mueve como un niño de su edad		*Tropieza, movimientos repetitivos, pobre coordinación o movimientos inusuales*		*Movimientos extraños de dedos, postura peculiar de dedos y cuerpo. Mirada fija. Autoagresión*		*Mayor intensidad y persistencia de las conductas extrañas (punto 3).*

V. Uso de objetos:

1	1.5	2	2.5	3	3.5	4
Interés en juguetes y otros objetos con los que juega apropiadament e		*Puede mostrar interés atípico o jugar de modo excesivamente infantil*		*Poco interés en juguetes o absorto en ellos. Fascinado por la luz de un objeto. Realiza movimientos repetitivos.*		*Mayor intensidad y frecuencia de las conductas descritas en el punto 3.*

VI. Adaptación al cambio:

1	1.5	2	2.5	3	3.5	4
Respuesta apropiada a los cambios		*Persistencia en la actividad o en los mismos objetos aún con intervención del adulto*		*Resistencia activa a los cambios de rutina (enfado o tristeza)*		*Reacciones severas al cambio (furia o falla de cooperación)*

VII. Respuesta visual:

1	1.5	2	2.5	3	3.5	4
Respuesta visual adecuada		*Olvida mirar objetos o más interesado en ver espejos o luces. Cierta evitación de la mirada*		*Mirada fija, evita la mirada. Mira objetos desde un ángulo inusual. Sujeta los objetos muy cerca de sus ojos*		*Evita resistentemente la mirada de la gente. Conductas extremas del punto 3*

VIII. Respuesta auditiva:

1 1.5 2 2.5 3 3.5 4

Respuestas auditivas adecuadas		***Puede haber falta de respuesta o reacción ligeramente extrema a ciertos sonidos***		***Respuesta variante. Ignora a menudo un sonido. Asustarse o cubrirse los oídos aún siendo sonidos conocidos***		***Puede reaccionar de modo extremo o no reaccionar a sonidos frecuentes***

IX. Gusto, olfato y uso y respuesta táctil:

1 1.5 2 2.5 3 3.5 4

Uso normal de los sentidos		***Persistencia en ponerse objetos en la boca. Puede oler o probar objetos no comestibles. Puede ignorar o reaccionar de modo extraño a dolores suaves***		***Puede estar moderadamente absorto tocando, oliendo o probando objetos, o a personas. Puede reaccionar mucho o muy poco a estímulos sensoriales***		***Absorto oliendo, probando o sintiendo objetos más por la sensación que por la exploración. Puede ignorar el dolor o sobrerreaccionar a un ligero malestar***

X. Ansiedad y miedo:

1 1.5 2 2.5 3 3.5 4

Conducta adecuada para ambas situaciones		***Ocasionalmente muestra excesivo o muy poco miedo o ansiedad comparado con sus iguales.***		***Muestra más o menos miedo de lo que sería típico de un niño más joven en situación similar***		***Sus miedos persisten tras una experiencia repetida con sucesos u objetos inofensivos. Es muy difícil de calmar. Puede fallar en percibir peligros que otros niños de su edad eviten***

XI. Comunicación verbal:

1 1.5 2 2.5 3 3.5 4

Comunicación verbal adecuada		***Retraso global del habla. Mayor parte del habla tiene poco significado. Puede haber repetición o inversión de pronombres. Usa palabras peculiares o jerga***		***Puede haber ausencia de habla. Si habla, puede haber mezcla de lenguaje con sentido y lenguaje peculiar (repetición o inversión de pronombres)***		***Sin uso del habla con significado. Gritos infantiles, sonidos extraños o parecidos a animales. Puede usar palabras o frases reconocibles.***

XII. Comunicación no verbal:

1 1.5 2 2.5 3 3.5 4

Comunicación no verbal apropiada para su edad		***Uso inmaduro. Puede señalar vagamente (peor que los niños de su edad)***		***Generalmente incapaz de expresar necesidades o deseos sin hablar. No entiende la comunicación no verbal de los otros***		***Sólo utiliza gestos extravagantes o peculiares, aparentemente sin significado. No capta gestos o expresiones faciales de otros***

XIII. Nivel de actividad:

1 1.5 2 2.5 3 3.5 4

Nivel de actividad normal para su edad		***Puede ser movido o un poco vago y lento. Nivel de actividad interfiere ligeramente en su funcionamiento***		***Bastante activo y difícil de frenar. Puede tener energía ilimitada, con dificultades para dormir en la noche. A la inversa, muy aletargado y necesita mucho esfuerzo para moverse.***		***Exhibe extremos de actividad y puede cambiar de un extremo al otro.***

XIV. Nivel y consistencia de la respuesta intelectual:

1 1.5 2 2.5 3 3.5 4

Inteligencia normal		*No es tan brillante como los niños de su edad. Habilidades retardadas en todas las áreas*		*En general, menos brillantes que sus iguales. Puede acercarse a un funcionamient o normal en una o más áreas*		*En general menos brillante que sus iguales. Puede funcionar mejor que un niño normal de su edad en una o más áreas*

XV. Impresiones generales:

1	1.5	2	2.5	3	3.5	4
No muestra ninguno de los síntomas característico s de autismo		*Muestra sólo algunos síntomas o grado leve de autismo*		*Muestra un número de síntomas o un grado moderado de autismo*		*Muestra muchos síntomas o un grado extremo de autismo*

Puntaje Total :
15 – 29 : No Autista.
30 – 36 : Autista Medianamente Moderado.
37 – 60 : Autista Severo

EVALUADORA: …………………………………………………………………….
FECHA DE EVALUACIÓN: …………………………………………………….
INFORMANTE: ……………………………………………………………………

<u>PUNTAJE</u> **<u>AREA</u>**

I.	REFERENTE A LA GENTE	
II.	IMITACIÓN	
III.	RESPUESTA EMOCIONAL	
IV.	USO DEL CUERPO	
V.	USO DE OBJETOS	
VI.	ADAPTACIÓN AL CAMBIO	
VII.	RESPUESTA VISUAL	

VIII. RESPUESTA AUDITIVA

IX. RESPUESTA AL SABOR , OLOR, TACTO Y USO

X. MIEDO O NERVIOSISMO

XI. COMUNICACIÓN VERBAL

XII. COMUNICACIÓN NO VERBAL

XIII. NIVEL DE ACTIVIDAD

XIV. NIVEL Y CONSISTENCIA DE LA RESPUESTA INTELECTUAL

XV. IMPRESIONES GENERALES

SUMATORIA TOTAL

DIAGNÓSTICO : ……………………………………………………...……..

Annex 11: Recommendations for parents or primary caregivers for collecting stool samples

RECOMENDACIONES PARA LAS MADRES Y PADRES PARA LA TOMA DE MUESTRA DE HECES

Es muy importante que lea atentamente estas recomendaciones antes de la toma de muestra:

Recibirán un frasco limpio estéril y una espátula o abatelenguas de madera para hacer la recogida de la muestra.

Para tomar la muestra, por favor siga los siguientes pasos:

1. Procure que la niña o el niño defeque en un recipiente limpio y seco (por ejemplo orinal), **evitando que las heces se mezclen con orina, papel higiénico o agua.**

   Es importante que el recipiente esté limpio y seco y no contenga restos de jabones, detergentes o desinfectantes.

   Si no dispone de orinal, o la niña o el niño es reticente a cambiar el lugar donde hace sus necesidades, puede realizar la recogida de muestra de la siguiente manera:

   OPCIÓN 1: Colocando papel de cocina recubriendo todo el fondo del inodoro,

   OPCIÓN 2: Colocando film transparente cubriendo toda la parte superior del inodoro

   OPCIÓN 3: Colocando una bolsa de plástico, de forma tal que se sostenga con el asiento.

   El niño o la niña se sentará al revés para que las heces no caigan al fondo del inodoro.

   Las heces caen sobre el papel de cocina o el plástico y no directamente al inodoro.

   En el caso de niños o de aquellos participantes que lleven pañales, las heces pueden recogerse directamente del pañal.

2. Se toma una **porción de heces del tamaño de una nuez** (aprox. 5 gramos) con la espátula o abatelenguas limpio y se introducen en el tubo o frasco. Cierre el frasco enroscando bien el tapón.

3. Escriba la **fecha y hora** de la recogida de la muestra.

4. **Coloque la muestra en hielo o en la refrigeradora a una temperatura de al menos 4ºC inmediatamente** después de recogerla.

5. Entregue a la persona responsable de la recogida lo más rápido posible, **antes de que pasen 12 horas** desde la toma de la muestra.

Annex 12: Supplementary information about gut microbiota analysis

1. DNA isolation

To characterize the microbiota, four steps will be followed: Stool samples will be transferred to a ZR bashingbead™ Lysis tube (Zymo Research, Catalogue No. S6012) containing 0.1 and 0.5 mm silica zirconium beads, followed by the addition of 750 µl of BashingBead™ buffer (Zymo Research, Catalogue No. D6001-3). These tubes will subsequently be homogenized in a vortex mixer for 40 min to disrupt the bacterial cell wall according to the manufacturer's recommendations. Total gDNA will be isolated using the Quick-DNA Fecal/Soil Microbe Miniprep Kit (Zymo Research, Catalogue No. D6010) following the manufacturer's procedure. Quality and quantity of isolated gDNA will be determined using the Nanodrop spectrophotometer (Thermo Fisher Scientific).

2. Library Preparation

The microbial gut (MG) composition will be analyzed using high-throughput Illumina sequencing of the hypervariable V4 region of the 16S rRNA gene. Library preparation will be constructed by amplifying the V4 region through two PCR reactions. The first PCR reaction (PCR I) will attach the Illumina adapter overhang sequences with the primer pair 16S_341F/16S_517R (Sravan *et al.*, 2017), yielding a 210 bp expected product size. The PCR I mixture will contain 1 ng to 1 µg gDNA, 1X Master Mix OneTaq Hot Start in Standard Buffer (New England BioLabs Inc., Catalogue No. M0484), 0.2 µM of each forward and reverse primer, and PCR-grade water to a final volume of 10 µL. The reaction will be conducted using SimpliAmp Thermal Cycler (Applied BiosystemsTM) with the following thermocycling programme: pre-denaturation at 94°C for 5 min, followed by ten cycles of 94°C for 15 s, 50-55°C for 30 s and 68°C for 30 s, and a final extension at 68°C for 10 min. The second PCR reaction (PCR II) wil further incorporate the index adapters i5 and i7 (MacConaill *et al.*, 2018). The PCR II mixture will contain 1 µL of PCR I amplicons, 5 µL OneTaq Hot Start 2X Master Mix in Standard Buffer (New England BioLabs Inc., Catalog No. M0484), 0.5 µL of 10 µM of i5 and i7 index primers, and 3 µL PCR-grade water, following the manufacturer's instructions. This reaction will be carried out using the same thermal cycler with the following thermocycling programme: pre-denaturation at 95°C for 5 min, followed by thirty cycles of 95°C for 15 s, 50-55°C for 30 s and 68°C for 30 s, and a final extension at 68°C for 5 min. Final PCR products will be cleaned-up using the AMPure XP Beads protocol (Agencourt Bioscience Corporation, Beverly, MA, USA) at 1X ratio. The concentration of the final sequencing libraries will be determined using Qubit 4 fluorometer (Thermo Fisher Scientific). Gel electrophoresis and Agilent 2100 Bioanalyzer System (Agilent Technologies, Palo Alto, CA) will be used to ensure correct product size.

3. Sequencing

Sequencing will be performed using an Illumina MiSeq Platform with paired-end reads of 300 bp, and V3 flowcell sequencing reagents, in a single run lasting approximately 65 hours. This will result in raw files in .FASTQ format with more than 100,000 reads per sample, which is recommended for taxonomic profiling (Liu *et al.*, 2020). Sequencing will be conducted in triplicate.

4. Bioinformatics

The DADA2 protocol for 16S rRNA metabarcoding analysis will involve several essential steps: loading and filtering raw sequence data, inferring exact sequence variants, and constructing a sequence variant table. Initially, raw sequences in FASTQ format will be imported and subjected to quality filtering to remove low-quality reads, typically using quality scores and length thresholds. Then, error rates will be modelled based on the quality scores of the sequences. Identical sequences will be deduplicated to minimize redundancy, and an algorithm will be employed to infer amplicon sequence variants (ASVs), distinguishing true biological sequences from sequencing errors. This process will include error correction and the resolution of unique variants. Finally, an ASV table will be generated, linking each inferred variant to its respective sample, providing a detailed profile of variant abundances across the dataset (Callahan *et al.*, 2016).

Annex 13: Informed consent to be signed by parents/legal guardians.

CONSENTIMIENTO INFORMADO PARA PADRES/MADRES/CUIDADORES PARA PARTICIPAR EN UN PROYECTO DE INVESTIGACIÓN SOBRE TEA

TÍTULO DEL PROYECTO: Disfunción de la modulación sensorial y su asociación con malnutrición y diferencias en las características de la microbiota intestinal en niños/as con trastorno del espectro autista - Proyecto Wiñay: un estudio transversal comparativo

Este estudio está financiado por la Escuela Superior Politécnica de Chimborazo y ha sido aprobado por el Comité de Ética en Investigación en Seres Humanos de la Escuela Superior Politécnica De Chimborazo (CEISH-ESPOCH). La investigadora principal es la Dra. Tannia Valeria Carpio Arias, docente investigadora de la ESPOCH.

¿CUÁL ES EL PROPÓSITO DE ESTE ESTUDIO?

El propósito de este estudio es determinar la asociación entre alteraciones sensoriales, malnutrición y diferencias en las características de la microbiota intestinal en niños/as y adolescentes ecuatorianos con TEA (trastorno del espectro autista) en comparación con niñas/os y adolescentes sin TEA y niñas/os y adolescentes con otros trastornos del desarrollo diferentes de TEA. El objetivo final es comprender cómo las alteraciones sensoriales afectan la alimentación de los niños con TEA y ésta en la flora intestinal. Este conocimiento podrá guiar a padres y a educadores en la adaptación de entornos y estrategias que faciliten la integración sensorial y el desarrollo saludable de estos niños, contribuyendo a su inclusión social y bienestar general.

¿EN QUÉ CONSISTE LA PARTICIPACIÓN EN EL ESTUDIO?

En total se organizarán tres reuniones de una duración aproximada de 60 minutos cada una, con las niñas y niños participantes y sus padres, madres, o cuidadores principales. La duración y número de sesiones sin embargo se adaptará a las necesidades de los cuidadores, las niñas y los niños.

En la primera sesión, explicaremos el procedimiento del estudio y le realizaremos una entrevista, en la que recopilaremos información sociodemográfica y sobre la salud y la alimentación de su hija/o. También se realizará un cuestionario de recordatorio de 24 horas en el que usted describirá lo que el niño comió el día anterior a la visita/entrevista. Llenar este cuestionario tardará aproximadamente 15 a 20 minutos. Adicionalmente, algunos padres y madres serán invitados a participar en una entrevista individual en profundidad para conocer los hábitos alimentarios y las preferencias alimentarias de sus hijos/as.

A las madres, padres o personas que pasan mayor tiempo con las/los niñas/os se les pedirá que llenen un cuestionario llamado POSI en el caso de bebés, y AQ-10 en el caso de niños y adolescentes, que sirve para evaluar cómo las guaguas se relacionan con la familia y amigos, lo que llamamos habilidades sociales. Como parte de la misma evaluación las investigadoras van también a observar cómo las guaguas se relacionan con otras personas y con lo que tienen alrededor, esta evaluación se llama CARS.

En el segundo encuentro se medirá el peso, talla y cantidad de grasa y agua del cuerpo de las niñas y niños participantes, lo que hará a través de una báscula electrónica especial llamada báscula de bioimpedancia y una cinta métrica. Para la toma de estas medidas se contará con

un lugar adecuado (escuela, hogar, etc) y se tomarán bajo la supervisión de usted como padre/madre de familia o cuidadora. Para la toma de estas medidas, el/la niño/niña requerirá estar con ropa cómoda y sin medias para el uso de la balanza de bioimpedancia. Si la niña o el niño presenta rechazo o resistencia, se suspenderá la toma de la información.

Al final de cada sesión, las niñas y los niños participarán en una nueva App llamada BlinkLab, que mide el parpadeo y otras reacciones a estímulos sonoros mientras la niña o el niño está mirando un video de su elección. Su hija/o mirará algunos videos cortos en la pantalla del dispositivo y la cámara del teléfono grabará la reacción de su hija/o. Las niñas/os participarán en dos videos de unos 15-20 minutos cada uno. Los videos que verá su hija/o durante el estudio fueron diseñados para ser alegres o neutrales para los niños pequeños, se supone que ninguno de ellos debería angustiar o asustar a su hija/o. No se le dará una devolución sobre la evaluación de la App y de este video. Su contribución al desarrollo de esta App es importante porque podría a futuro contribuir a desarrollar nuevas técnicas diagnósticas para personas con TEA.

Las imágenes y sonidos (videos, fotografías u otros medios) grabados serán utilizados exclusivamente en este estudio para realizar una codificación y evaluación de los comportamientos observados. Estas grabaciones quedarán almacenadas bajo seguridad en nuestros servidores de datos con protección bajo password. Los datos obtenidos serán tratados, con respeto a la vigente normativa de protección de datos, de forma seudonimizada. Sus datos nunca se cederán a terceros salvo obligación legal. Puede ejercitar sus derechos en relación con el tratamiento de sus datos personales de acceso, rectificación y supresión, entre otros, mediante solicitud dirigida a la persona responsable de la investigación.

Estas evaluaciones podrán extenderse según el protocolo y las necesidades de los participantes. Las pruebas que no se consigan realizar en la segunda sesión se podrán programar en una tercera sesión. A lo largo del periodo de evaluación, los padres y madres podrán contactar con los investigadores para resolver sus dudas. Si lo prefieren, los padres y madres podrán responder a los cuestionarios en casa, ya que pueden ser auto-administrados en en el hogar para luego ser entregados al personal investigador.

También recogeremos muestras de heces de las niñas y niños, con su colaboración, que usaremos para analizar la composición de la flora intestinal. Las personas responsables del estudio le proporcionarán un documento donde se detalla cómo se debe recoger la muestra. Las muestras recolectadas serán tratadas confidencialmente, sólo serán usadas para este estudio y almacenadas en un ultra-congelador mientras dure el estudio. Una vez finalizado el mismo, las muestras serán eliminadas mediante incineración. En ningún caso usaremos métodos invasivos y respetaremos en todo momento la voluntad de continuar o no en la investigación. El rechazo o resistencia de su hija/o a colaborar (manifestada verbal o no verbalmente por signos de malestar, ansiedad, etc.) supondrá la interrupción inmediata de la tarea o/y en caso de desear continuar, la búsqueda de condiciones o momentos de evaluación más oportunos.

Con la firma de este documento, usted también autoriza a que los investigadores consulten la historia clínica de su hija/o para que podamos contrastar y confirmar datos sobre la salud, las terapias y los medicamentos que han sido recetados a su hija/o en los últimos meses.

Toda la información que usted proporcione será tratada de manera confidencial. Sus respuestas serán anónimas y no se asociará con su nombre en ningún informe o publicación. Usted puede decidir dejar de participar en el estudio en cualquier momento sin dar ninguna explicación. Esto no afectará de ninguna manera a la atención que recibe su hija/o.

Existe la posibilidad de que le enviemos avisos a su dispositivo o dirección de correo electrónico para pedirle que complete las actividades y las encuestas en su dispositivo. Puede elegir hacerlo en el momento que a Ud. le convenga. Ocasionalmente, nos volveremos a poner en contacto con Ud. para pedirle que participe en nuevas secciones del proyecto, o para que provea comentarios y sugerencias adicionales.

¿CUÁL ES EL RESULTADO FINAL DE ESTE ESTUDIO?

Los resultados de este estudio contribuirán a mejorar nuestros conocimientos sobre las niñas, niño y adolescentes con TEA en el Ecuador. Los datos que obtengamos en este estudio contribuirán a que puedan desarrollarse nuevos tratamientos, nuevos métodos diagnósticos, y también para que puedan implementarse políticas que mejoren la calidad de vida de las personas con TEA.

¿RECIBIRÉ ALGUNA COMPENSACIÓN POR PARTICIPAR?

Ningún participante recibirá compensación económica por su participación. La participación tampoco tiene ningún costo para las y los participantes. Al finalizar el estudio, las profesionales en nutrición que trabajan en este estudio proporcionarán asesoramiento nutricional personalizado a las niñas y niños participantes. Adicionalmente, se organizará una sesión para todos los padres y madres participantes en la que se presentarán de forma sencilla y accesible los resultados generales del estudio. Sin embargo bajo ninguna circunstancia, en esta socialización se utilizarán datos que permitan identificar de manera personal a alguno de las y los participantes del estudio

¿CUÁLES SON LOS RIESGOS DEL ESTUDIO?

No se conocen riesgos físicos asociados a este estudio, ni para Ud. ni para su hijo/a. Un riesgo posible de este estudio es la incomodidad que algunas personas sienten cuando contestan preguntas relacionadas con cuestiones personales o emocionales. Algunas de las preguntas que le haremos sobre Ud. o su hija/o pueden hacerle sentir incómodo/a. **Puede negarse a contestar cualquiera de las preguntas, y puede tomarse un descanso en cualquier momento** durante el estudio.

Al participar en los cuestionarios sobre salud, usted o su hija/o podrían recibir valoraciones diagnósticas que quizá no esperen y que pueden ser indeseables o perturbadoras. El personal investigador hará todo lo posible por respetar su intimidad y ofrecer soporte emocional e información en todo momento que se requiera.

Existe el riesgo de que se produzcan filtraciones de datos a terceros de manera indeseada. Nuestro equipo de investigadoras hará todo lo posible para que esto no suceda, codificando sus datos y guardándose todos los datos siempre bajo contraseña, a la que tendrán acceso solo las personas involucradas en el estudio.

DERECHOS Y OPCIONES DE LOS PARTICIPANTES

La participación en este estudio es completamente voluntaria. Al aceptar que **los datos personales y las muestras de heces fecales** del participante sean utilizados con fines de investigación, no se renuncia a ninguno de los derechos que por ley les **pertenecen.** La información contenida en los datos personales o la información que se genere del análisis de

las muestras biológicas humanas (heces fecales) de los participantes serán utilizadas únicamente para este fin y nunca se colocarán o publicarán datos que permitan revelar su identidad, debido a que toda la información se anonimizará (codificará) con la finalidad de respetar la confidencialidad.
El participante será libre de retirar el consentimiento en cualquier momento, para lo cual deberá informar al investigador principal Tannia Valeria Carpio, a través del contacto 0995807568 y correo electrónico tannia.carpio@espoch.edu.ec. En ese momento los datos obtenidos serán eliminados y no podrán ser utilizados para ningún otro fin. Esto no causará ninguna penalidad ni tendrá impacto alguno en la atención en salud que por ley le corresponde al niña/niño participante.

CONFIDENCIALIDAD DE DATOS: Toda la información recabada será anonimizada de la siguiente manera: se usará códigos alfanuméricos que no permitan la identificación personal de las participantes o que contengan información personal (e.g., fecha de nacimiento, género, dirección de domicilio, correo electrónico, etc.).
Dicho código tendrá el siguiente formato ddmmaaaa-nnnn, en el cual dd corresponde a día del mes en números, mm corresponde a mes del año en números, aaaa corresponde a año y nnnn corresponde al orden en que se tomaron los datos. Por ejemplo, el día 11 de septiembre del 2022, María Emilia participa en el estudio en primer lugar, luego José Tomas y finalmente Guillermo. El código para Emilia sería 11092022-0001, el código para José Tomas sería, 11092022-0002 y el de Guillermo sería 11092022-0003.

Solo el cuestionario de características demográficas(Anexo 1.1) incluirán datos personales para poder codificar. El resto de instrumentos de recolección de datos incluirá en el encabezado el código correspondiente a cada participante, lo que se usará con la finalidad de poder conectar los instrumentos o documentos entre sí. La transcripción de los datos a la base de datos electrónica se realizará usando el código alfanumérico como identificador. No se transcribirá los datos personales de los participantes que se recogen en el Anexo 1.1. A dicha información solo tendrá acceso la directora del proyecto y personal autorizado. La información personal al igual que los documentos físicos serán almacenados y resguardados por la Directora del proyecto y personal autorizado. Una vez finalizado el estudio y haya transcurrido el tiempo legalmente establecido se procederá a destruir los datos personales que corresponden a los códigos de los participantes.

INFORMACIÓN DE CONTACTO: En cualquier momento usted puede comunicarse con la institución donde reposan o almacenan la información de su representado como datos personales y muestras biológicas humanas (heces fecales), para que a su vez sirva como canal de comunicación con los investigadores que hagan uso de la información de salud de su representada/o. Para lo cual, puede comunicarse a los siguientes teléfonos 0995807568 y correo electrónico tannia.carpio@espoch.edu.ec.

DECLARATORIA DE CONSENTIMIENTO INFORMADO:
Yo____ (nombres completos del sujeto/representante legal de (colocar los nombres completos de la representada/o): ____), comprendo que mis datos personales y muestras biológicas humanas (heces fecales) de mi representado/a serán utilizados con fines de investigación científica cuyo objetivo previamente me fue explicado. Me han explicado los riesgos y beneficios de la utilización de los datos de mis datos personales y muestras biológicas humanas (heces fecales) de mi representado/a en un

lenguaje claro y sencillo. Han respondido a todas las preguntas que he realizado y me entregaron una copia de este documento. Entiendo que en todo momento los investigadores tomarán las medidas necesarias para precautelar la confidencialidad de los datos personales y muestras biológicas humanas (heces fecales) de mi representada/o.

Entiendo que los datos confidenciales serán utilizados exclusivamente para la investigación científica propuesta, y solo eventualmente para investigaciones científicas posteriores relacionadas con la misma línea de investigación, para las que se otorgue explícitamente y en su momento, un nuevo consentimiento informado escrito previo a la aprobación del protocolo respectivo por un Comité de Ética de Investigación en Seres Humanos reconocido por el Ministerio de Salud Pública. En virtud de lo cual, voluntariamente (Marque con una X):

ACEPTO

NO ACEPTO

Nombres completos del sujeto /representante legal

____ Cédula de ciudadanía/

pasaporte del sujeto/representante legal __________________________________

Firma/huella digital del sujeto/representante

legal____

Fecha y lugar ____

Declaratoria de revocatoria del consentimiento informado

YO,____, representante legal de ___________________________________, de forma libre y consciente he decidido retirar el consentimiento de participación en esta investigación de mi hijo/hija/representado.

Lugar y Fecha:____

FIRMA DEL
PARTICIPANTE:____

Annex 14: Informed consent in accessible language to be signed by adolescents aged 15 and older.

Asentimiento para adolescentes a partir de 15 años en lenguaje accesible para participar en el proyecto de investigación "WIÑAY"

HOJA DE INFORMACIÓN PARA MENORES

TÍTULO DEL PROYECTO: Disfunción de la modulación sensorial y su asociación con malnutrición y diferencias en las características de la microbiota intestinal en niños/as con trastorno del espectro autista - Proyecto Wiñay: un estudio transversal comparativo

Estamos realizando una investigación para saber más sobre el TEA (trastorno del espectro autista), y te pedimos que nos ayudes participando en este estudio. Por favor, lee esta hoja antes de decirnos lo que piensas. Puedes preguntar todo lo que quieras a tus padres, a otros familiares, o a nosotros mismos, y tomarte el tiempo que quieras antes de decirnos si quieres participar o no.

¿Por qué te estamos pidiendo que participes?
Queremos saber más cosas sobre el TEA (trastorno del espectro autista) e investigar las diferencias de los sentidos, malnutrición y diferencias en la microbiota intestinal en niños/as y adolescentes ecuatorianos con TEA. Nuestro objetivo final es mejorar la salud de niños, niñas y adolescentes con TEA.

Tu participación es libre, si no quieres participar no tienes por qué hacerlo. También es importante que sepas que si decides participar ahora y más adelante, en cualquier momento, ya no quieres seguir en el estudio, puedes decirnos que has cambiado de opinión y decirnos que ya no quieres participar, sin tener que dar explicaciones. En ese caso no habrá ningún problema, tus consultas y los cuidados que necesites continuarán igual.

¿Qué tendrás que hacer?
Si participas en este estudio,

- Haremos algunas preguntas a tus padres o cuidadores sobre ti y tu estado de salud.
- Mediremos tu peso, talla y cintura usando una báscula, infantómetro o tallímetro y cinta métrica
- Te haremos algunas preguntas a ti.
- Con la ayuda de tus padres o cuidadores tomaremos una muestra de heces (caca) para analizarla.
- Usaremos una App llamada BlinkLab que graba tus reacciones mientras tú estás viendo una película en el celular.

No tendrás ninguna molestia durante el estudio, y no hay ningún riesgo para tu salud. Si en cualquier momento te sientes incómodo/a, puedes no contestar a cualquiera de las preguntas, y no pasará nada. También puedes tomarte un descanso en cualquier momento, solo tienes que avisarnos.

Participar en este estudio nos ayudará a saber más cosas sobre el TEA, y probablemente ayude en el futuro a otros niños/as y adolescentes que tengan TEA.

Información sobre ti y el estudio:

La información que se guarde sobre ti y todos los datos sobre tu salud, incluido si tienes TEA o no, serán un secreto. Sólo las personas que están realizando el estudio sabrán quién eres. No se guardará tu nombre, sino que toda la información sobre ti se guardará con un código, y solo los investigadores sabrán que ese código te corresponde a ti. Mientras dure el estudio, tus muestras de heces serán almacenadas en un ultra-congelador. Cuando se acabe el estudio, tus muestras serán eliminadas.

Los y las investigadoras publicarán los resultados de este estudio en revistas científicas, pero nadie sabrá que has participado ni cuáles fueron tus resultados.

Información adicional y datos de contacto
Si tienes cualquier pregunta sobre el estudio, puedes ponerte en contacto o pedirle a tus padres que se pongan en contacto con los investigadores, preguntando a: Dra. Valeria Carpio (Teléfono 0995807568 y correo electrónico tannia.carpio@espoch.edu.ec)

Si quieres participar, por favor, pon una X en el cuadrito de abajo que dice "Sí quiero participar" y escribe tu nombre.

□ Sí quiero participar

□ NO quiero participar

Nombre: ___________________________

Annex 15: Informed consent for children and adolescents with verbal communication difficulties, for use by research staff.

ASENTIMIENTO INFORMADO PARA NIÑAS, NIÑOS y ADOLESCENTES CON DISCAPACIDAD DEL APRENDIZAJE NO VERBAL

ASENTIMIENTO INFORMADO PARA NIÑAS, NIÑOS y ADOLESCENTES CON DISCAPACIDAD DEL APRENDIZAJE NO VERBAL

PRIMERA SESIÓN

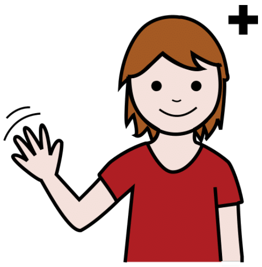

¡HOLA!

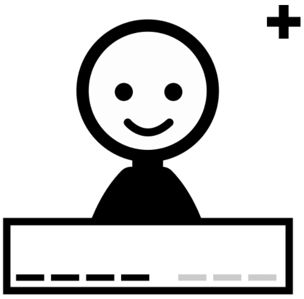

Nuestro nombre es ___________________________

Te invitamos a participar en nuestro proyecto.

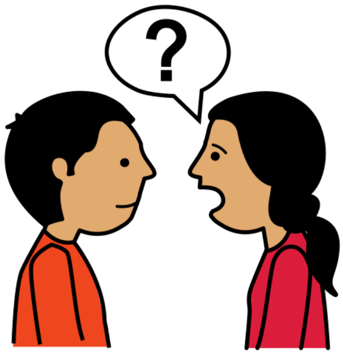

Haremos algunas preguntas.

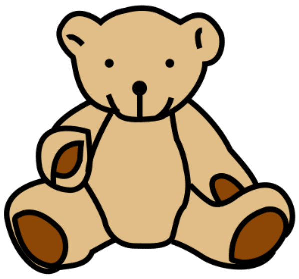

Puedes traer tu peluche favorito

Si necesitas una PAUSA podemos parar. Sólo tienes que decir “PAUSA” o señalar aquí te dejaremos descansar el tiempo que quieras.

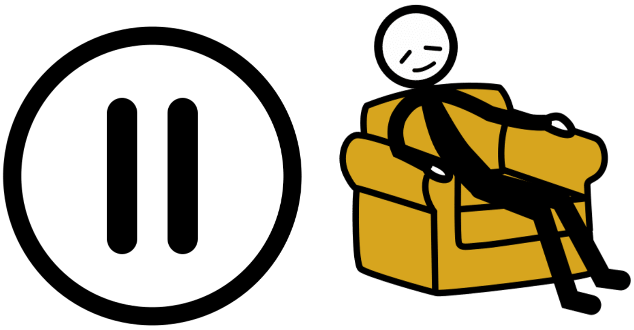

¿Quieres participar?

SI NO

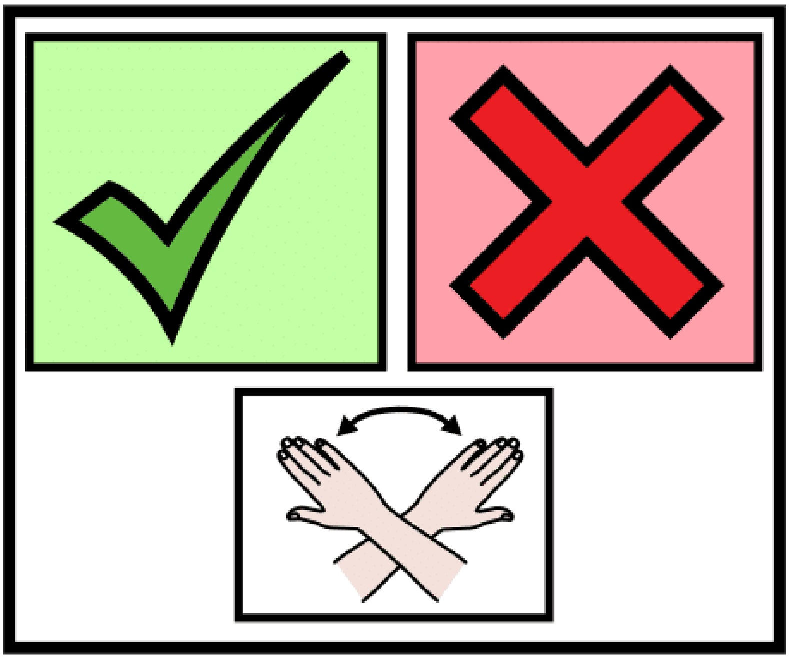

SEGUNDA SESIÓN

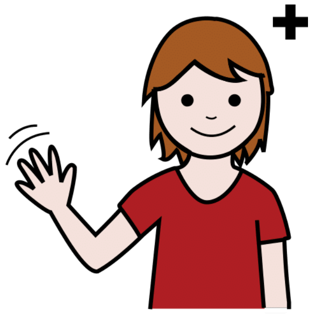

¡HOLA DE NUEVO!

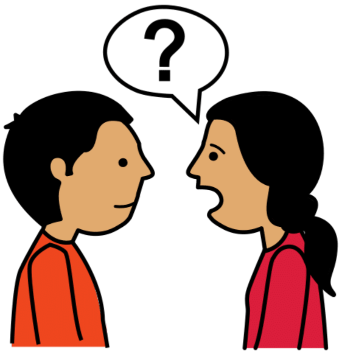

Hoy haremos algunas preguntas,

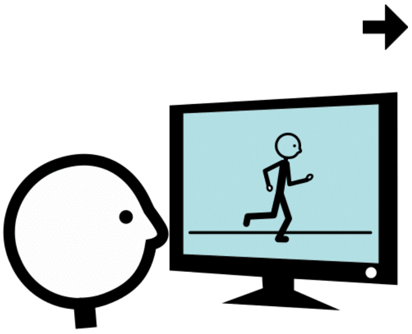

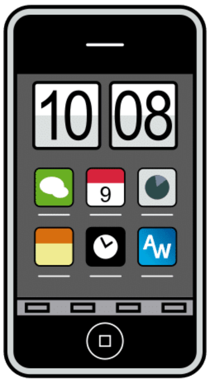

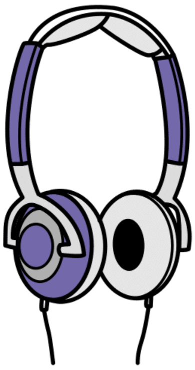

Miraremos películas en el celular.

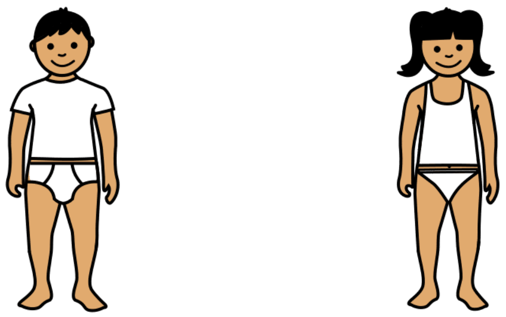

Te quitaremos la camiseta, el pantalón, medias y zapatos

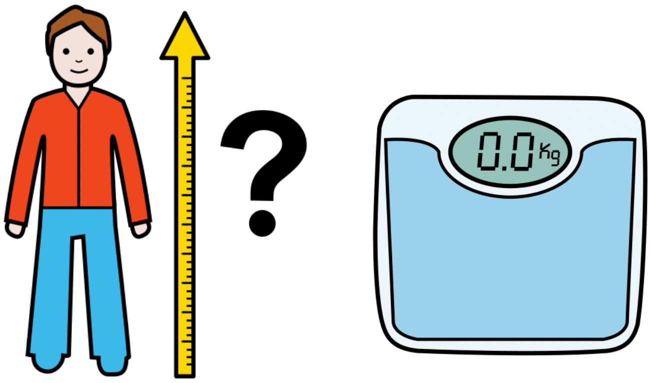

Para medir tu peso y altura

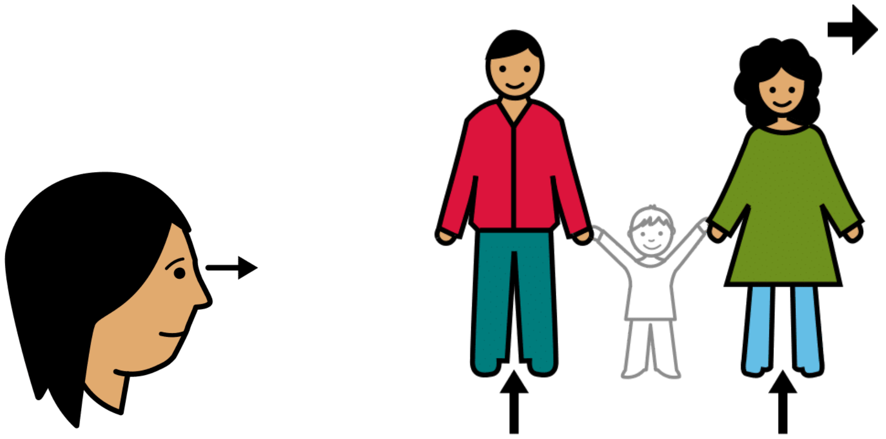

Tu madre o padre estarán presentes durante la medición

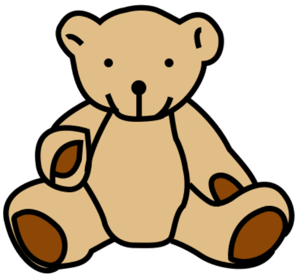

Puedes traer a tu peluche favorito.

Si necesitas una PAUSA podemos parar. Sólo tienes que decir “PAUSA” o señalar aquí te dejaremos descansar el tiempo que quieras.

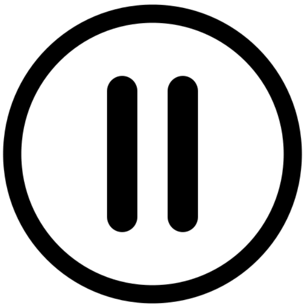

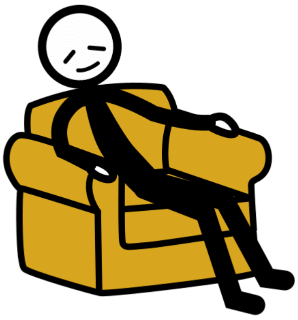

¿Quieres participar?

SI NO

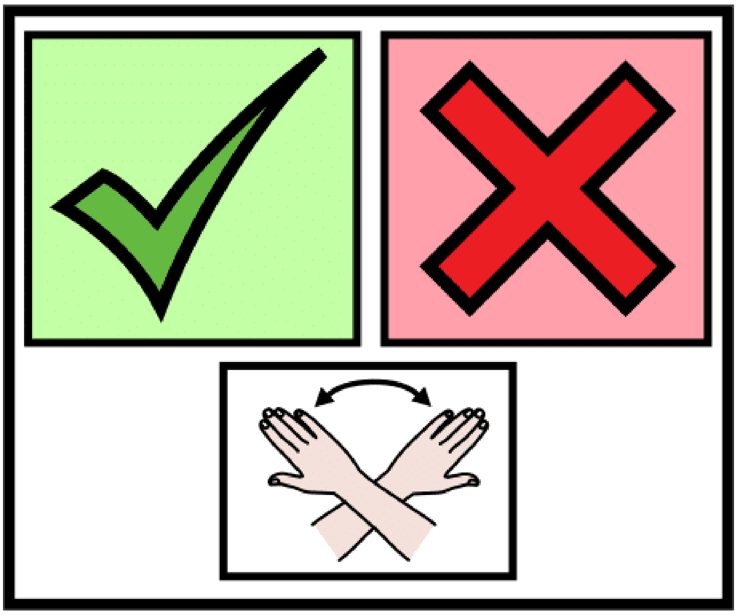

TERCERA SESIÓN:

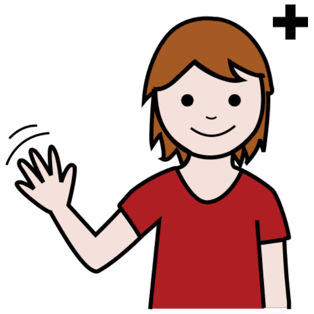

¡HOLA DE NUEVO!

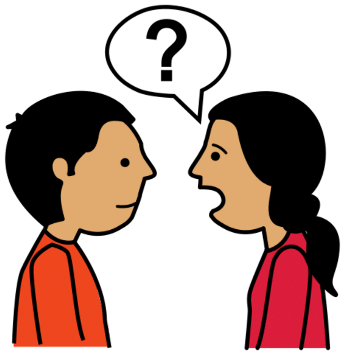

Hoy haremos algunas preguntas,

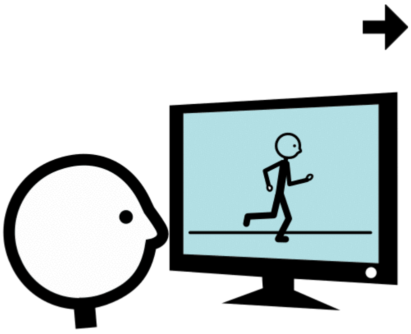

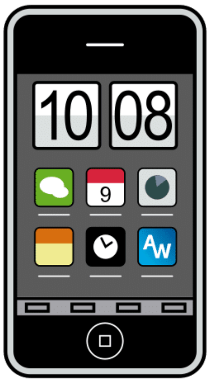

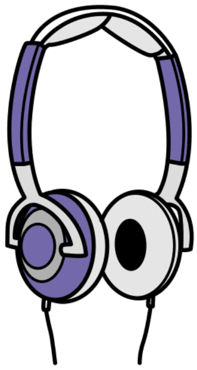

Miraremos películas en el celular.

Si necesitas una PAUSA podemos parar. Sólo tienes que decir "PAUSA" o señalar aquí te dejaremos descansar el tiempo que quieras.

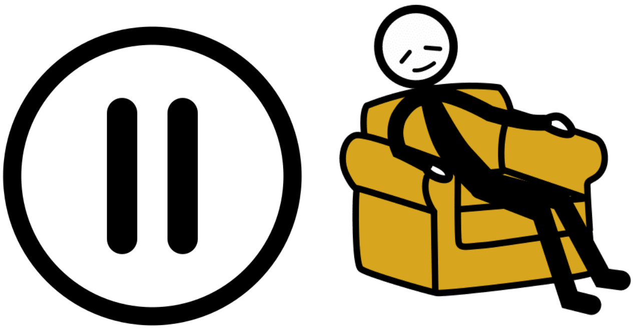

¿Quieres participar?

SI NO

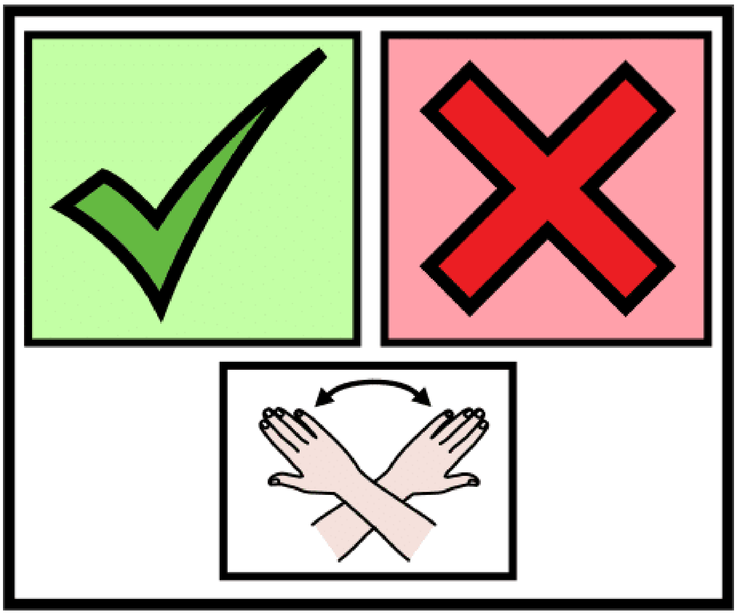

The pictographic symbols used are the property of the Government of Aragon and were created by Sergio Palao for ARASAAC (http://www.arasaac.org), which distributes them under a Creative Commons BY-NC-SA license.

2. Supplementary Figures and Tables

2.1 Supplementary Figure

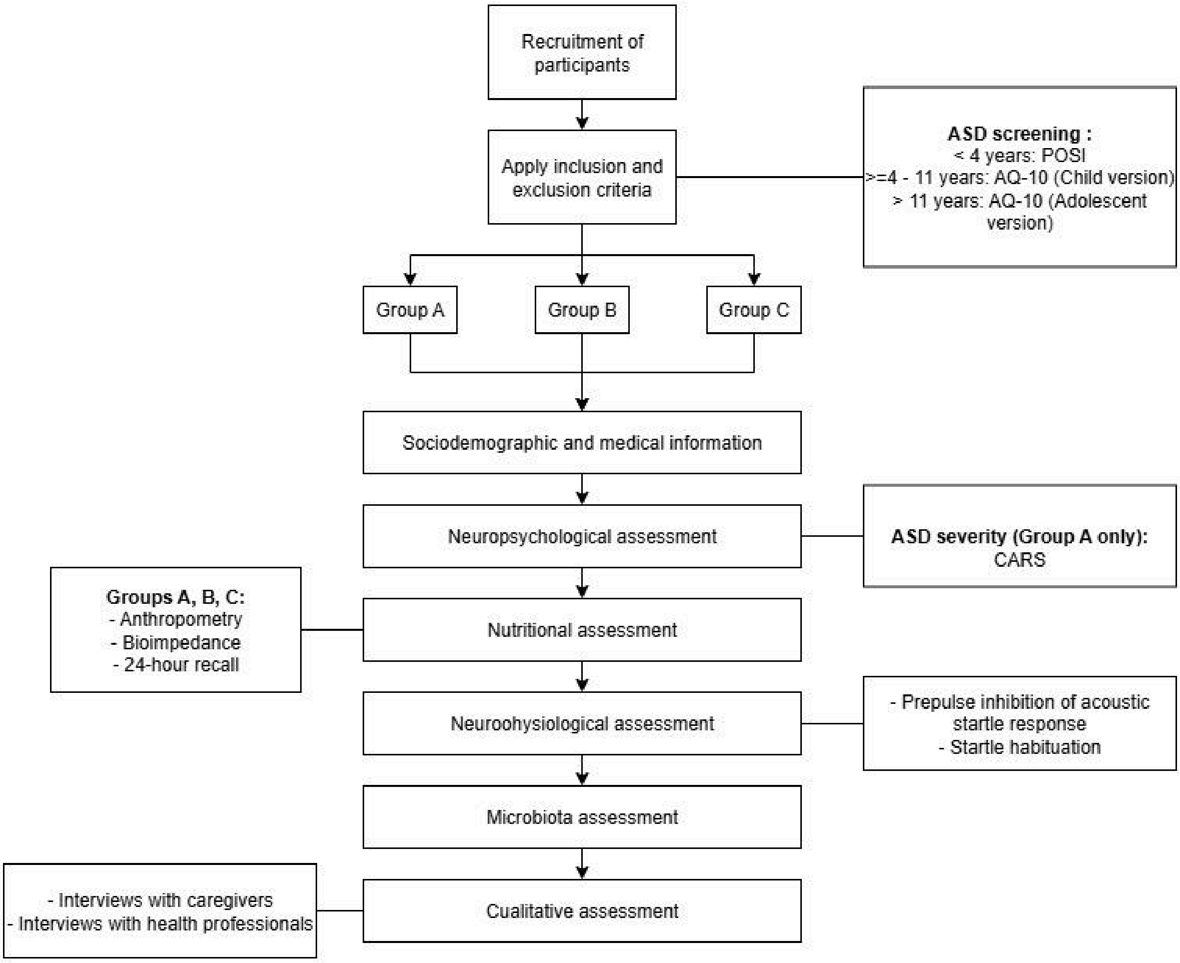

Supplementary Figure 1. Flow diagram of the "Proyecto Wiñay" study procedures.
AQ-10 – 10-item Autism Spectrum Quotient; ASD – Autism Spectrum Disorder; CARS – Childhood Autism Rating Scale; POSI – Parent's Observations of Social Interactions.
